# Tuning surface properties of thiophene-based thin films on glass substrates for cancer cell adhesion, drug release control, and computational analysis

**DOI:** 10.1038/s41598-025-05691-w

**Published:** 2025-06-20

**Authors:** Heba M. Metwally, Omar M. El-Banna, Ehab Abdel-Latif, Raghda Abo Gabal

**Affiliations:** 1 Department of Chemistry, Faculty of Science, Mansoura University, Mansoura, 35516 Egypt; 2 Center of Excellence for Genome and Cancer Research, Urology and Nephrology Center, Mansoura University, El Dakhlia, Egypt

**Keywords:** Thiophene, Thin film glass, Hepatocellular, Thin film, Cancer adhesion, Drug release, Docking, DFT, Biophysics, Cancer, Computational biology and bioinformatics, Chemistry, Materials science, Nanoscience and technology, Physics

## Abstract

This study explores the potential of six novel thiophene derivative thin films (THIOs) for reducing cancer cell adhesion and enhancing controlled drug release on inert glass substrates. Thiophene derivatives **3a–c** and **5a–c** were synthesized and characterized using IR, ^1^H NMR, ^13^C NMR, and elemental analysis before being spin-coated onto glass to form thin films. SEM analysis and roughness measurements were used to assess their structural and functional properties. Biological evaluations demonstrated that the films significantly reduced HepG2 liver cancer cell adhesion (~ 78% decrease vs. control) and enabled controlled drug release, validated through the Korsmeyer-Peppas model (R^2^ > 0.99). Theoretical studies, including in-silico target prediction, molecular docking with JAK1 (PDB: 4E4L), and DFT calculations, provided insights into the electronic properties and chemical reactivity of these compounds. Notably, compound **5b** exhibited the best binding energy (-7.59 kcal/mol) within the JAK1 pocket, aligning with its observed apoptotic behavior in cell culture. DFT calculations further revealed that **5b** had the lowest calculated energy values; -4.89 eV (HOMO) and − 3.22 eV (LUMO), and the energy gap was found to be 1.66 eV, supporting its role in JAK1 inhibition and cancer cell adhesion reduction. These findings underscore the promise of thiophene derivatives in biomedical applications, potentially leading to safer surgical procedures and more effective localized drug delivery systems.

**Supplementary Information:**

The online version contains supplementary material available at 10.1038/s41598-025-05691-w.

## 1. Introduction

Surgical tools used for tumor biopsy, resection, or ablation are susceptible to cancer cell adherence when they come into direct contact with tumor tissue, which can cause complications. These attached cancer cells may stay viable and spread to surrounding healthy tissues, potentially triggering the development of new tumors^1,2^. Cancer seeding along the needle tract has been notably observed in patients undergoing percutaneous needle biopsy (PNB) for breast and metastatic colorectal cancers^3,4^. Intriguingly, concerning data reveal that 26.7% of patients with primary or secondary liver tumor exhibit proliferative cancer cells on the ablation needles used during RFA procedures, explaining the 12.5% incidence of cancer seeding along the needle tract in RFA for primary liver cancer^5^. To address this challenge, researchers have explored various interventions^6^.

The tendency of surgical instruments to facilitate cancer cell adhesion highlights the importance of understanding and mitigating potential risks, particularly in liver cancer, bladder cancer, and renal cell carcinoma treatments. Although tumor seeding along the needle tract is uncommon, it has been documented in cancers such as breast, metastatic colorectal, and papillary renal cell carcinoma, with renal biopsies exhibiting an incidence of less than 1%. Risk factors, including multiple needle passes and the use of larger gauge needles, may contribute to this phenomenon. Similarly, while bladder cancer biopsies are rare due to the preference for transurethral resection, isolated cases of tumor seeding have been reported. Investigating the application of THIO coatings in these cancers could provide valuable insights into their potential to minimize cell adhesion and improve surgical safety across various tumor environments^7^. Expanding research to include bladder cancer, renal cell carcinoma, and other cancer types such as breast (MCF-7, MDA-MB-231)^8^, colorectal (HCT116, SW480)^9^, lung (A549, H1975)^10^, prostate (LNCaP, PC-3)^11^ and ovarian (SKOV3)^12^ cancer could help evaluate the broader efficacy of these coatings, predominantly in avoiding tumor cell adhesion during biopsy and surgical procedures.

Radiofrequency ablation (RFA) emerges as a crucial therapeutic approach for health care recipients with unresectable liver malignancies. However, alarming findings indicate that a substantial proportion of patients 26.7% of those with primary or secondary liver malignancies-harbor proliferative cancer cells exposed on the ablation needles used during RFA procedures^13,14^. This revelation raises a critical concern: the potential for cancer cell dissemination and seeding during RFA, leading to disease recurrence and complications^15^. Understanding the mechanisms underlying cancer cell adhesion to surgical instruments and their impact on patient outcomes is imperative. Cancer cell attachment is influenced by hydrophobicity, roughness, stiffness, topography, charge, and curvature^16,17^. The interplay of these factors shapes adhesion strength and specificity, impacting drug delivery, biomaterials, and targeted therapies^18,19^. By unraveling these mechanisms, researchers and clinicians can devise strategies to mitigate the risk of cancer cell dissemination and enhance the efficacy of liver cancer treatments such as RFA^20,21^. Chitosan films incorporating a 2-aminothiophene derivative exhibited antifungal effects against *Candida* species^22^. Alternative materials have been more extensively studied, such as a thermosensitive anti-adhesive agent based on a human-derived acellular dermal matrix^23^, which showed promise in preventing postoperative adhesions. Similarly, visible-light curable furfuryl gelatin derivatives loaded with ibuprofen have demonstrated effectiveness as biocompatible, drug-loaded anti-adhesion barriers^24^.

Heterocyclic compounds shows unlimited significance in both medicinal and organic chemistry because of their extensive biological activities. Compounds containing sulfur, nitrogen, and oxygen in their structures consistently attract the interest of medicinal chemists and researchers due to their wide-ranging pharmacological properties^25–31^. One such heterocycle is thiophene, a five-membered ring with a sulfur heteroatom. Thiophene-containing compounds have been extensively studied for their potential as pharmaceutical agents, demonstrating a wide range of pharmacological activities^32,33^. For many years, thiophene derivatives, including mono-substituted, di-substituted, and tri-substituted forms, have been identified as effective anticancer drugs. Several anticancer drugs currently on the market incorporate a thiophene nucleus in their composition. Thiophene-based coatings generally exhibit stability under physiological conditions, including variations in pH and temperature. Quinoid-thiophene-based covalent organic polymers have demonstrated resilience even under conditions harsher than room temperature^34^. However studies have shown that thiophene-containing photosensitizers can react with singlet oxygen, leading to potential degradation^35,36^.

These activities include antimicrobial^37,38^, antifungal^39^, antiviral^40^, antitumor^41^, anti-inflammatory^42^, and antioxidant^43^ properties. Thiophene derivatives have attracted interest in cancer research because of their capacity to affect several cellular processes related to tumor growth. When conjugated with folate, thiophene-based compounds can specifically target these receptors^44^. In particular, some tetrahydrobenzo[b]thiophene derivatives function as destabilizers of tubulin polymerization, inhibitors of histone deacetylase (HDAC), and blockers of topoisomerases^45^. These varied mechanisms emphasize the adaptability of thiophene derivatives in cancer treatment, providing various pathways for targeted therapeutic approaches.

Many available thiophene based drugs exhibiting anticancer action through multiple pathways involved in cancer^46–57^. **Raloxifene**, thiophene analogue, is commonly used in reduction risk of invasive breast cancer in post-menopausal women^58^. Also, Tyrosine Kinase Inhibitor **OSI-930** for both tumor cell proliferation and tumor angiogenesis in a variety of cancers^59^. **Teniposide**, is used in childhood acute lymphocytic leukemia treatment protocol^60^. Positron emission tomography, PET-imaging of inflammation biomarkers were used to track metabolic processes radiolabeled with thiophene^61^. Scientists and researchers aim to design and develop innovative, target-specific, and advanced thiophene derivatives. Thiophene derivatives can be engineered to enhance drug delivery systems, enabling targeted therapies for cancer by directing nanoparticles to specific cells^45^. Introducing polar groups (e.g., –COOH or –X) carboxy or halogeno derivatives could improve hydrophilicity and binding to cancer-specific receptors^62^.

The tumor microenvironment plays a critical role in drug resistance and chemotherapy success^63,64^. Partly by influencing signaling pathways such as JAK/STAT, which regulate cancer cell adhesion, proliferation, and migration. Inhibiting this pathway can alter adhesion dynamics^65^. Certain thiophene compounds have demonstrated the ability to inhibit vascular endothelial growth factor receptor 2 (VEGFR-2) and AKT, key proteins involved in cancer cell proliferation and survival^66^.

It was observed that apoptosis sensitivity increases by direct contact with specific extracellular matrix (ECM) components^67^. Previous research has underscored the critical role of surface topography, surface free energy, and chemical composition in determining cellular adhesion strength^68^. Therefore, our focus was on assessing the impact of thio-modified glass (THIOMG) surfaces specifically on cancer cell adhesion, as well as their potential for controlled drug release applications^69,70^. By selecting an inert glass substrate, we could isolate the effects of the THIOs and analyze their potential as biomedical coatings.

This research hypothesizes that thiophene-based (THIO) thin film coatings on glass substrates can effectively reduce cancer cell adhesion, enhance biocompatibility, and enable controlled drug release, making them promising candidates for biomedical applications, particularly in implantable medical devices and surgical instruments. Additionally, it is proposed that different THIO derivatives-methyl, methoxy, and chloride will exhibit distinct biological effects on cancer cells due to their varying electronic properties, influencing cell adhesion, viability, and proliferation^71^.

Six novel thiophene-based derivatives were chemically synthesized and characterized using spectroscopic methods. These compounds were spin-coated onto glass substrates, forming thin films, whose quality was assessed through scanning electron microscopy (SEM) and surface roughness analysis. Additionally, drug release kinetics were investigated to understand the controlled release behavior of these coatings. To evaluate the biomedical potential of these coatings, HepG2 liver cancer cells were seeded onto the THIO-coated glass substrates, allowing for the assessment of their cytotoxic effects on cancer cell adhesion, viability, and proliferation. Molecular docking studies were also conducted to determine the binding affinities and interactions of the synthesized THIO derivatives with key biomolecules associated with liver cancer, providing insights into their roles as potential inhibitors or modulators of cancer-related pathways.

## Materials and methods

### Chemistry experimental

All data related to the devices utilized in the chemistry section were included in the supplementary materials.

#### Synthesis of 3-hydroxythiophene compounds 3a–c

The 2-(arylhydrazono)-2-ethoxycarbonyl-thioacetanilide derivative **2a–c** was prepared as reported^72^ and (0.002 mol) of each was dissolved in sodium ethoxide solution (which was prepared from 0.04 g Na and 20 mL absolute ethanol). The mixture was stirred for 10 min then 2-chloro-*N*-(4-sulfamoylphenyl)acetamide (**1**) (0.510 g, 0.002 mol) was added. Then, the mixture was refluxed for 2 h at 80 °C and the formed precipitate on hot was dried out and crystallized from ethanol to give the targeted 3-hydroxythiophenes **3a–c**. These conditions were optimized based on prior studies^77^.

##### 3-hydroxy-5-(phenylamino)-N-(4-sulfamoylphenyl)-4-(p-tolyldiazenyl)thiophene-2-carboxamide (3a)

Black solid, yield = 64.1%, m.p. = above 300 °C. IR (ν/cm^− 1^): 3529, 3189 (NH & NH_2_), 1621 (C=O), 1325 and 1157 (SO_2_). ^1^H NMR (δ/ppm): 2.29 (s, 3 H, CH_3_), 6.99 (d, *J =* 7.5 Hz, 1H, Ar-H), 7.05–7.22 (m, 6 H, Ar-H&2NH), 7.28–7.47 (m, 5 H, Ar-H), 7.64–7.68 (m, 4 H, Ar-H&OH), 11.68&12.47 (s, 1H, *cis/trans* conformers NH), 14.59&15.03 (s, 1H, *cis/trans* conformers NH). ^13^C NMR (δ/ppm): 20.51, 90.71, 91.14, 112.42, 113.77, 115.44, 116.97, 120.50, 121.32 (2 C), 125.01, 126.89, 129.02, 129.26 (2 C), 129.95, 133.05, 135.25, 141.72, 144.14, 147.83, 157.79, 162.95, 167.09. Anal. Calcd. %for C_24_H_21_N_5_O_4_S_2_ (507.10): C, 56.79; H, 4.17; N, 13.80% Found: C, 56.50; H, 4.32; N, 13.65%.

##### 3-hydroxy-4-((4-methoxyphenyl)diazenyl)-5-(phenylamino)-N-(4-sulfamoylphenyl)thiophene-2-carboxamide (3b)

Black solid, yield = 61.1%, m.p. = above 300 °C. IR (ν/cm^− 1^): 3558, 3511, 3359 (OH& NH &NH_2_), 1634 (C=O), 1303 and 1154 (SO_2_). ^1^H NMR (δ/ppm): 3.77 (s, 3 H, OCH_3_), 6.93 (d, *J* = 9.5 Hz, 1H, Ar-H), 6.95–7.14 (m, 4 H, Ar-H&2NH), 7.18 (t, *J* = 7.25 Hz, 1H, Ar-H), 7.22–7.34 (m, 3 H, Ar-H), 7.41–7.48 (m, 3 H, Ar-H), 7.62–7.70 (m, 4 H, Ar-H&OH), 11.66&12.47 (s, 1H, *cis/trans* conformers NH), 14.38&15.05 (s, 1H, *cis/trans* conformers NH). Analysis calculated for C_24_H_21_N_5_O_5_S_2_ (523.10): C, 55.06; H, 4.04; N, 13.38% Found: C, 55.36; H, 4.19; N, 13.58%.

##### 4-((4-chlorophenyl)diazenyl)-3-hydroxy-5-(phenylamino)-N-(4-sulfamoylphenyl)thiophene-2-carboxamide (3c)

Black solid, yield = 73.6%, m.p. = 295 °C. IR (ν/cm^− 1^): 3422(OH), 3309, 3201, 3240 (NH&NH_2_), 1646 (C =O), 1304 and 1162(SO_2_). ^1^H NMR (δ/ppm): 7.00 (d, *J* = 7.5 Hz, 1H, Ar-H), 7.10–7.18 (m, 2 H, Ar-H), 7.21 (t, *J* = 7.25 Hz, 1H, Ar-H), 7.29–7.46 (m, 8 H, Ar-H& 2NH), 7.60–7.70 (m, 4 H, Ar-H&OH), 11.67&12.43 (s, 1H, *cis/trans* conformers NH), 14.55&15.06 (s, 1H, *cis/trans* conformers NH). Anal. Calcd. for C_23_H_18_ClN_5_O_4_S_2_ (527.05): C, 52.32; H, 3.44; N, 13.26% Found: C, 52.08; H, 3.59; N, 13.44%.

#### Synthesis of 3-methylthiophene compounds 5a–c

The 2-acetyl-2-arylazo-thioacetanilide derivatives **4a**-**c** was prepared as reported^72,77^ and (0.002 mol) of each was dissolved in sodium ethoxide solution (which was prepared from 0.04 g Na and 20 mL absolute ethanol). The mixture was stirred for 10 min then 2-chloro-*N*-(4-sulfamoylphenyl)acetamide (**1**) (0.510 g, 0.002 mol) was added. The mixture was refluxed for 2 h then the formed precipitate on hot was dried and crystallized from ethanol to give the targeted 3-methylthiophenes **5a–c**. These conditions were optimized based on prior studies^72,77^.

##### 3-methyl-5-(phenylamino)-N-(4-sulfamoylphenyl)-4-(p-tolyldiazenyl)thiophene-2-carboxamide (5a) 

Orange solid, yield = 62.9%, m.p. = above 300 °C. IR (ν/cm^− 1^): 3380&3270 (NH&NH_2_), 1642 (C=O), 1311 and 1154 (SO_2_). ^1^H NMR (δ/ppm): 2.31 (s, 3 H, CH_3_), 2.49 (s, 3 H, CH_3_), 7.20–7.32 (m, 2 H, Ar-H), 7.38 (d, *J =* 7.5 Hz, 2 H, Ar-H), 7.45–7.53 (m, 6 H, Ar-H&NH_2_), 7.65–7.80 (m, 4 H, Ar-H), 12.30 &13.04 (s, 1H, *cis/trans* conformers NH), 15.08&16.65 (s, 1H, *cis/trans* conformers NH). ^13^C NMR (δ/ppm): 13.01, 20.67, 116.90 (2 C), 120.12 (2 C), 120.82 (3 C), 125.78, 126.49 (2 C), 129.68 (3 C), 130.06 (2 C), 135.27, 135.71, 136.53, 138.66, 142.65, 146.40, 155.14, 161.50.Anal. Calcd. for C_25_H_23_N_5_O_3_S_2_ (505.12): C, 59.39; H, 4.59; N, 13.85% Found: C, 59.62; H, 4.70; N, 13.75%.

##### 4-((4-methoxyphenyl)diazenyl)-3-methyl-5-(phenylamino)-N-(4-sulfamoylphenyl)thiophene-2-carboxamide (5b)

Orange solid, yield = 45%, m.p. = 293–295 °C. IR (ν/cm^− 1^): 3364 and 3262 (NH&NH_2_), 1639 (C=O), 1311 and 1153 (SO_2_) .^1^H NMR (δ/ppm): 2.57 (s, 3 H, CH_3_), 3.81 (s, 3 H, OCH_3_), 7.05 (d, *J* = 9 Hz, 2 H, Ar-H), 7.24 (t, *J* = 7.25 Hz 1H, Ar-H), 7.41–7.55 (m, 5 H, Ar-H&2NH), 7.63–7.83 (m, 7 H, Ar-H), 10.15 (s, 1H, NH) 13.62 (s, 1H, NH). ^13^C NMR (δ/ppm): 13.32, 55.49, 114.75, 118.33, 120.04 (2 C), 120.20, 120.41 (3 C), 125.30, 126.54 (2 C), 129.75 (3 C), 134.24, 138.52, 138.64, 141.44, 142.10, 143.73, 151.21, 158.84, 161.46. Anal. Calcd. for C_25_H_23_N_5_O_4_S_2_ (521.61): C, 57.57; H, 4.44; N, 13.43% Found: C, 57.49; H, 4.59; N, 13.28%.

##### 4-((4-chlorophenyl)diazenyl)-3-methyl-5-(phenylamino)-N-(4-sulfamoylphenyl)thiophene-2-carboxamide (5c) 

Orange solid, yield = 71.4% %, m.p. = above 300 °C. IR (ν/cm^− 1^): 3374 and 3266 (NH&NH_2_), 1634 (C=O), 1312 and 1154 (SO_2_). ^1^H NMR (δ/ppm): 2.52 (s, 3 H, CH_3_), 7.26–7.90 (m, 15 H, Ar-H&2NH), 10.76 (s, 1H, NH) 14.15 (s, 1H, NH). Anal. Calcd. for C_24_H_20_ClN_5_O_3_S_2_ (525.07): C, 54.80; H, 3.83; N, 13.31% Found: C, 54.75; H, 3.98; N, 13.15%.

### Fabrication of thiophene-glass thin film derivatives

The six novel thiophene powders (**3a–c** and **5a–c**) were dissolved in DMSO at room temperature. Each thiophene derivative (55 mg) was individually dissolved in 5 mL of DMSO and filtered. Using the spin coating method, thiophene thin films were applied to cleaned optical glass substrates with a VTC100A Compact Spin Coater, set to 8000 rpm for 4-inch wafers. The speed of the spin coater was adjusted to 2500 rpm, and a drop of each solution was placed onto the rotating substrate to form the films. The films were allowed to dry for 48 h at room temperature in the dark.

### Characterization of thiophene-glass thin film derivatives

#### Field emission scanning electron microscopy (FE-SEM)

Field emission scanning electron microscopy (FE-SEM) using a JEOL JSM-IT100 (Japan) provided comprehensive information about the size and morphology of the deposited materials. To improve conductivity, three samples were coated with a thin gold layer (1.5–3 nm) at 30 kV and 30 µA. The surface roughness was assessed to analyze the synthesized surface profile on the PCL scaffolds. For quantitative evaluation, J-Image software was utilized to measure the arithmetical mean roughness (Ra), the root mean square roughness (Rq), Skewness (Rsk), Kurtosis (Rku), Maximum peak height (Rp), the lowest valley(Rz)^72,73^.

#### In vitro release studies

In vitro release studies for all samples were conducted in PBS (pH 7.4) with 0.2% Tween 80. Each sample was dispersed in 2 mL of the release medium contained within a Falcon tube holding 50 mL of PBS. At set time intervals, 2 mL was withdrawn and replaced with fresh media. After generating a standard curve, the concentration of the drug released from each sample was quantified using UV-Vis spectroscopy at the relevant wavelengths. The release kinetics were analyzed using various models, including Zero-Order Kinetics, First-Order Kinetics, the Higuchi Model, and the Korsmeyer-Peppas Model. A cumulative release curve was then created, plotting the percentage of drug released over time, with data collected in triplicate, and presented as mean ± standard deviation (SD).

#### Sterilization of materials for cell culture studies

For cell culture experiments, materials were organized as thin films and located at the underside of tissue culture plastic dishes. These films were sterilized by soaking in 75% ethanol for 10 min, followed by rinsing with PBS to remove any remaining alcohol. Finally, the films were sterilized using UV light.

#### Setting up cell cultures on experimental films

The study investigated the cytotoxic effects of different materials on HepG2 human liver cancer cells. **HepG2** cell lines were purchased from Nawah Scientific Company, Egypt. HepG2 cells were grown in DMEM high glucose medium supplemented with 10% fetal bovine serum (FBS) and antibiotics. The cells were seeded at a density of 2 × 10^4^ cells/cm^2^ in 3 ml of serum-containing medium on either material films as a control. After the incubation period, the culture plates were removed, and the medium was discarded. The glass surfaces were washed three times with phosphate-buffered saline (PBS) to remove any unattached or loosely adhered cells. Cytotoxicity was assessed by measuring the reduction in cell growth using J-image analysis, which involved capturing images to evaluate cell viability and proliferation^74^. Cell area measurements were conducted to assess the morphological changes of HepG2 cells in response to various treatment conditions. The cells were cultured under standard conditions and divided into several groups: an untreated control group (HepG2) and six treatment groups labeled 3a, 3b, 3c, 5a, 5b, and 5c. The cell area was quantified through Python utilizing libraries skimage. A minimum of 100 cells per group were analyzed to ensure statistical reliability, and the experiment was repeated across independent replicates to validate the consistency of the observed trends. To optimize data accuracy, image preprocessing steps including grayscale conversion, background subtraction, and noise filtering were applied. These procedures allowed for a clear and reliable assessment of cell morphology, ensuring robust data for subsequent statistical analysis.

### Theoretical calculations

#### In silico-target prediction

In Silico-Target Prediction was performed using https://www.swisstargetprediction.ch made by Swiss Institute of Bioinformatics. For determining the target-affinity prediction of our compounds to specific kinase enzyme, computational Tools for Analyzing PPI and Chemico-Protein Interactions was used. The primary sources of data incorporated into STRING include experimental interaction data, computationally annotated complexes and pathways from existing databases, automated text mining from scientific literature, genomic context predictions, and evidence transferred from other organisms, accessible at https://string-db.org/. The interaction scores provided by STRING range from 0 to 1, with 1 indicating the highest confidence level. Additionally, the PPI networks generated by STRING can be visualized using the Cytoscape plaThioorm (https://cytoscape.org/), facilitating a comprehensive understanding of the interaction landscape.

#### Molecular docking

To study the binding capabilities of the target compounds, Molecular Operating Environment (MOE, 2019.01) software was exploited for docking purpose. Refer to the Supporting Information for further details. The crystal structure of **JAK1 Gene-Janus Kinase 1** was downloaded from Protein Data Bank (PDB: 4E4L). Docking simulation of the synthesized compounds at JAK1 kinase active site was carried out in comparison with Jak1 gene protein codes downloaded from protein data bank (PDB: 3EYG, 3EYH, 4E4N and 4E5W).

#### DFT-calculation

The compounds were subjected to DFT simulations to verify the intended geometries of the molecules under inquiry. The DFT calculations were carried out utilizing the DMol^3^/BIOVIA-MS platform^75^ via GGA/RPBE functionality and the DNP basis set. DFT parameters were obtained by employing HOMO and LUMO values of energy to represent the chemical reactivity of compounds^76–79^.

#### Statistical analysis

ImageJ 64-bit software was utilized to quantify roughness results. Data are presented as mean ± standard deviation (SD). For Microscopic images, Statistical analysis was conducted using Python (version 3.12.7). To compare the control group with all treated groups (3a, 3b, 3c, 5a, 5b, 5c), the Kruskal-Wallis test (non-parametric method) was applied. The results indicated a statistically significant difference among the groups (*p* < 0.05), suggesting that at least one treatment group exhibited a distinct cell area distribution compared to the others. To explore differences between the control group and each individual treated group, Mann–Whitney U tests were performed. These pairwise comparisons confirmed statistically significant reductions in cell area variability across all treated groups relative to the control (*p* < 0.05), in agreement with the patterns observed in the boxplot visualizations. Results were expressed as medians with interquartile ranges (IQR).

## Results and discussion

### Chemistry

**Scheme 1:**
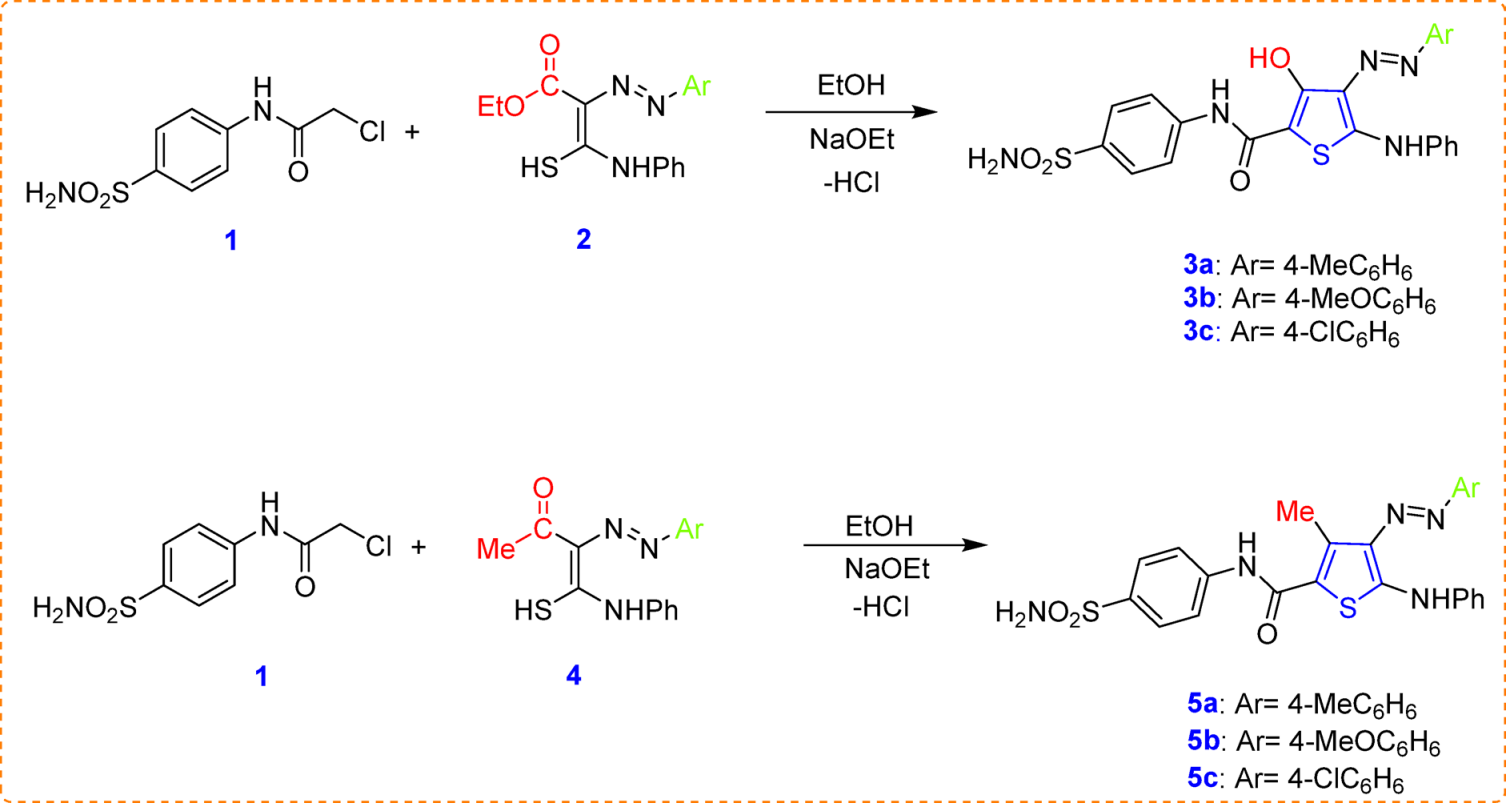
Synthesis of the novel 3-hydroxy thiophenes **3a–c** and 3-methyl thiophenes **5a–c**.

The targeted novel thiophene derivatives **3a–c** and **5a–c** were synthesized using a one-step process. This involved condensing 2-chloro-*N*-(4-sulfamoylphenyl)acetamide (**1**) with different functionalized thiocarbamoyl compounds **2** and **4**, respectively. The reported method has been used to prepare the multipurpose chloroacetamide compound, 2-chloro-*N*-(4-sulfamoylphenyl)acetamide (**1**)^80^. In an ethanolic hot sodium ethoxide solution, 2-chloro acetamide **1** was reacted with 2-(arylhydrazono)-2-ethoxycarbonyl-thioacetanilide (**2a–c**)^43,81^ (Scheme 1). Spectroscopic methods like IR, ^1^HNMR, ^13^CNMR and mass spectroscopy of the newly synthesized compounds were entirely in agreement with the designated molecular structures. Synthesis of our intended thiophene derivatives **5a–c** was carried out using the aforesaid successful one step approach. Thus, the reaction of 2-chloro acetamide **1** with 2-acetyl-2-arylazo-thioacetanilide derivatives (**4a–c**)^82,83^ in ethanol containing sodium ethoxide gave the corresponding 3-methyl thiophene-2-carboxamide derivatives **5a–c** (Scheme 1). The newly synthesized compound’s structures were confirmed using IR and ^1^H NMR data, which validated the assigned molecular structures.

### Characterization of thiophene-glass thin film derivatives

#### Field emission scanning electron microscopy (FE-SEM)

**Fig. 1:**
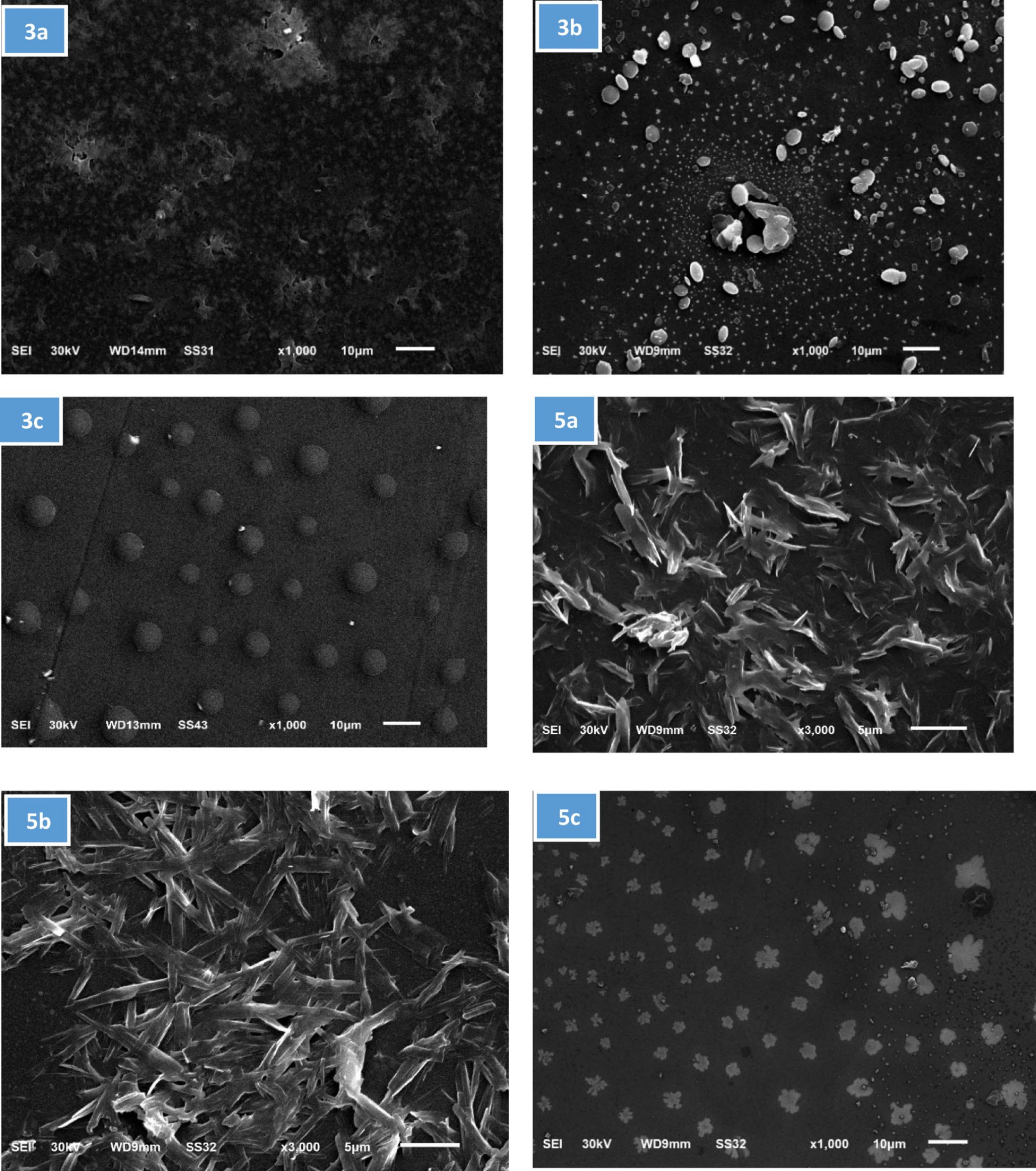
Scan electron microscope images for samples **3a–c**, **5a–c** (1000× magnification) showing surface morphology of thin films prepared via spin coating.

As shown in (Fig. 1), the SEM images demonstrate distinct differences in the surface morphology of thin films. In Image **3a**, Fig. 1, the surface is irregular and uneven, with large aggregates and voids, indicating poor uniformity and inconsistent deposition. Image **3b**, Fig. 1, shows a more uniform distribution of spherical particles, though there is a noticeable central cluster, possibly caused by localized defects or impurities during the spin-coating process. In Image **3c**, Fig. 1, the surface is the most uniform and smooth, with evenly distributed spherical particles and minimal defects, suggesting better control over the deposition process. This image represents the best thin film formation, featuring a smooth and consistent surface.

Similarly, the SEM images (Fig. 1 and **5a**, **5b**, and **5c**) highlight noticeable differences in the thin films surface morphology produced by spin coating. Figure 1 and **5a** and **5b**, exhibit fibrous, flaky structures with irregular shapes and random orientations, reflecting poor uniformity in the deposition process. Both images display elongated particles, but **5b** has a denser and more interwoven fiber arrangement, indicating a slight improvement in uniformity compared to **5a**. In contrast, Fig. 1 and 5c shows evenly distributed flower-like structures, indicating better control over the spin coating process and leading to higher-quality thin film formation.

**Fig. 2:**
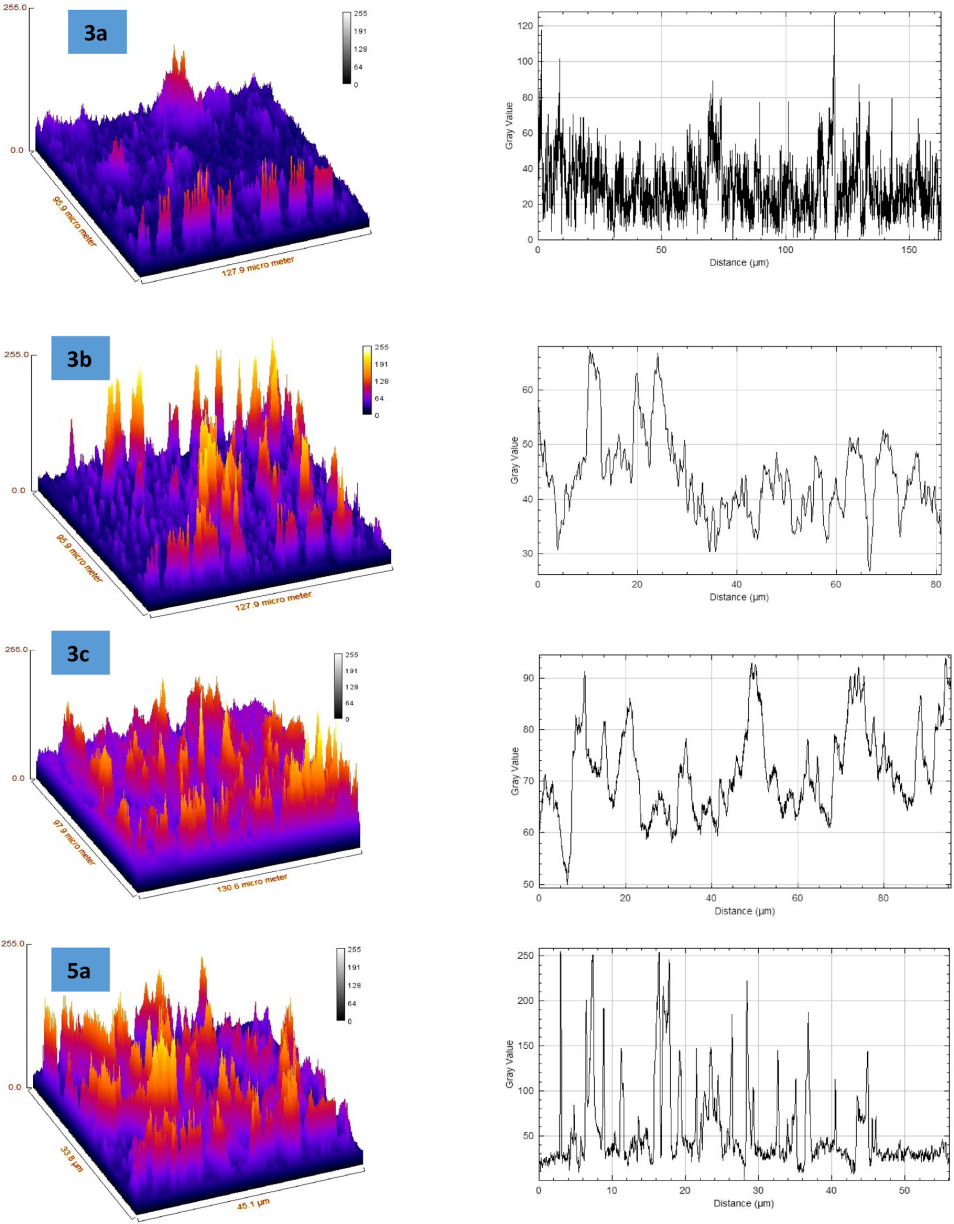

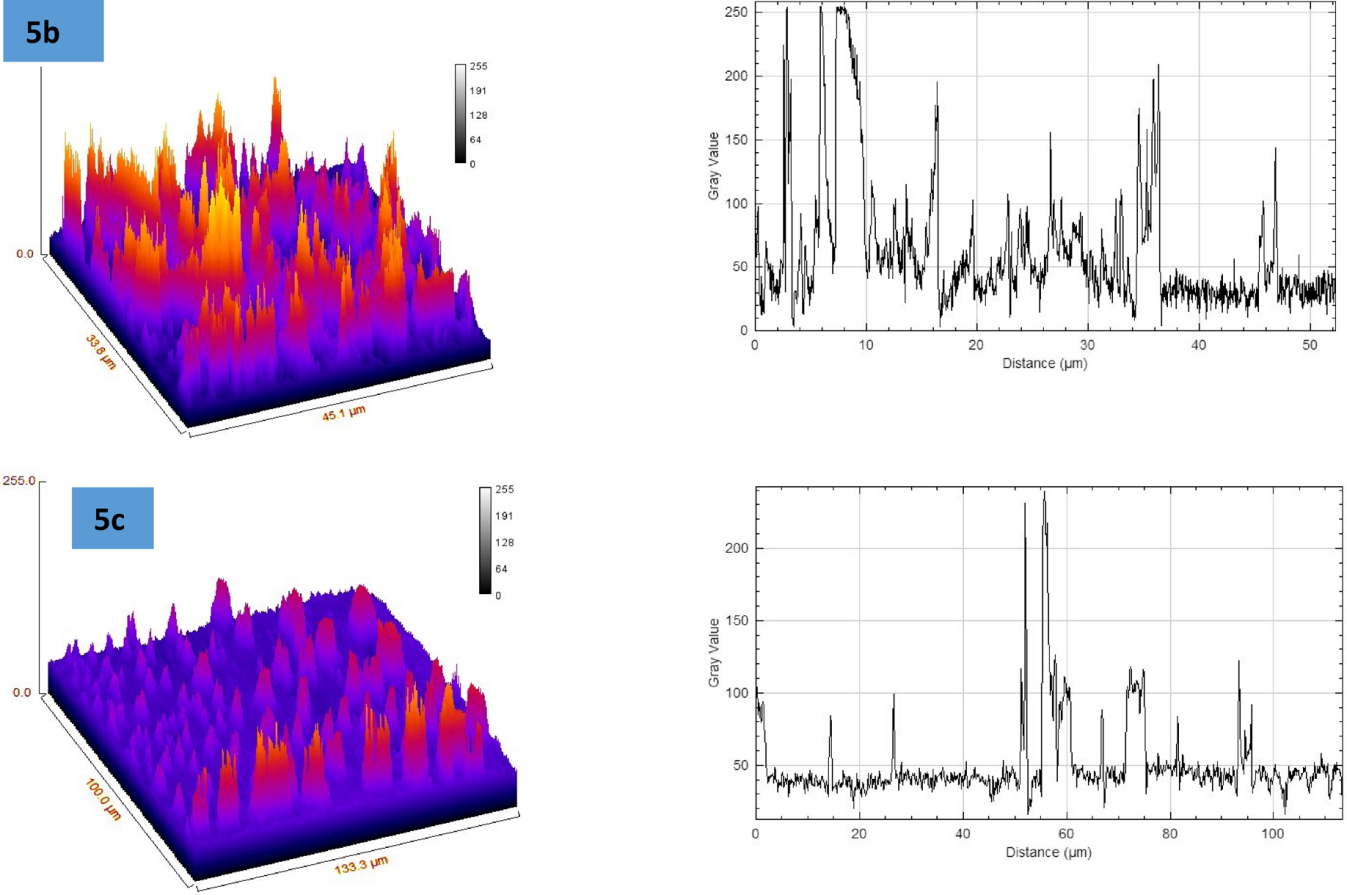
Surface plot and surface roughness curves for samples **3a–c** and **5a–c**.

**Table 1:** Surface roughness parameters for samples **3a-c** and **5a-c**, showing root mean square roughness (Rq), average roughness (Ra), skewness (Rsk), kurtosis (Rku), maximum peak height (Rp), the lowest valley(Rz).The table reflects the differences in surface texture, sharpness, and peak distribution across the samples.

Sample	Rq	Ra	Rsk	Rku	Rp	Rz
**3a**	25.5783	16.2791	3.9777	28.8427	255	0
**3b**	42.2802	24.7138	3.0804	9.7554	255	0
**3c**	38.01	27.29	1.60	4.61	255	0
**5a**	40.97	28.14	2.44	6.82	255	0
**5b**	43.61	31.07	1.20	4.88	255	0
**5c**	21.36	11.32	5.12	39.59	255	0

The roughness figures presented in the Fig. 2 reveal significant variation in surface topography across the samples with is corresponding surface profile curves, providing insight into the texture and features of each surface. The structural variations in the thiophene-2-carboxamide derivatives **3a–c** and **5a–c** play a crucial role in determining their behavior during thin-film formation on glass substrates. Compound **3a**, with its hydroxyl group and p-tolyl diazenyl substitution, exhibits rough surface morphology with large aggregates, indicating poor uniformity, likely due to limited interaction with the glass substrate and irregular solvent evaporation. In contrast, **3b** and **3c**, also featuring hydroxyl groups, demonstrate stronger hydrogen bonding with the substrate, leading to better adhesion and more uniform films. This is particularly evident in **3c**, where the presence of a chlorine atom adds a hydrophobic character but still allows for smoother deposition due to a balanced interaction between polar and non-polar regions. The 3-methyl-substituted compounds **5a–c** show reduced adhesion due to the absence of the hydroxyl group, resulting in rougher surfaces and less uniform film coverage. Among these, **5c**, with a chlorine substitution, displays improved uniformity compared to **5a**, likely due to the electron-withdrawing nature of the chlorine atom. Overall, the presence of hydrophilic groups (as in **3b** and **3c**) enhances film smoothness, while hydrophobic substitutions (as in **5a** and **5b**) result in rougher, less uniform thin films. The balance between polarity, hydrophobicity, and functional groups defines the quality and morphology of the thin films formed via spin coating.

As shown in Table 1 the surface roughness analysis of samples **3a–c** and **5a–c** reveals distinct characteristics across the set. Sample **3a** demonstrates moderate roughness, with an Rq of 25.58 and Ra of 16.28, and a strongly positive skewness (Rsk = 3.98) indicating a surface with more peaks, along with sharp features as reflected by its high kurtosis (Rku = 28.84). Sample **3b**, with a higher Rq (42.28) and Ra (24.71), is rougher than **3a**, but with less extreme skewness and kurtosis, though it maintains similar peak height. Sample **3c** has similar roughness to **3b** but with lower skewness (Rsk = 1.60) and kurtosis (Rku = 4.61), suggesting fewer sharp features. Sample **5a** shows comparable roughness to **3b** and **3c**, though it has sharper features than **3b**, as indicated by its kurtosis (Rku = 6.82). Sample **5b** is the roughest of all (Rq = 43.61, Ra = 31.07), with a moderate skewness and kurtosis. In contrast, sample **5c** has the lowest roughness values (Rq = 21.36, Ra = 11.32) but exhibits the most extreme skewness (Rsk = 5.12) and kurtosis (Rku = 39.59), indicating a surface with sharp, tall peaks. All samples exhibit high maximum peak heights (Rp = 255), suggesting significant variations in surface topography across the set.

#### In vitro release studies

**Table 2:** The fit parameters for different mathematical models (zero-order, first-order, higuchi, and Korsmeyer-Peppas models) used to analyze drug release from thin films.

Compounds	Zero order fit			First order fit			Higuchi fit				Korsmeyer-Peppas fit		
	K_0(µg.h_^−1^_)_	Intercept	*R* ^2^	K_1(h_^−1^_)_	Intercept	*R* ^2^	K_H(µg.h_^−1/2^_)_	Intercept	*R* ^2^	*n*	Intercept	Kp	*R* ^2^
**3a**	17.81	111.80	0.96	0.22	6.44	0.86	569.01	1247.70	0.93	0.59	1.38	23.99	0.99
**3b**	51.37	39.72	0.94	0.19	8.00	0.92	1515.36	3414.65	0.92	0.54	1.37	23.44	0.99
**3c**	0.44	20.97	0.74	0.15	2.77	0.92	49.15	87.73	0.97	0.85	1.37	23.44	0.99
**5a**	8.3	422.54	0.88	0.19	6.09	0.95	969.64	1712.27	0.97	0.88	1.38	23.99	1
**5b**	12.1	569.06	0.88	0.20	6.53	0.97	1331.62	2364.58	0.97	1.13	-1.57	0.027	0.99
**5c**	10.26	17.48	0.94	0.19	6.35	0.94	258.86	612.63	0.91	2.39	-3.31	0.001	0.99

Table 2 presents the fit parameters obtained from zero-order, first-order, Higuchi, and Korsmeyer-Peppas models for drug release profiles of various thin films labeled as **3a–c** and **5a–c**. The goodness-of-fit for each model is indicated by the R^2^ values. The intercept represents the initial burst release or initial conditions of the model. A more negative intercept suggests a higher initial burst or a deviation from the ideal linear model at early times. The highest R^2^ value for each model signifies the best fitting model for the respective thin film.

**Fig. 3:**
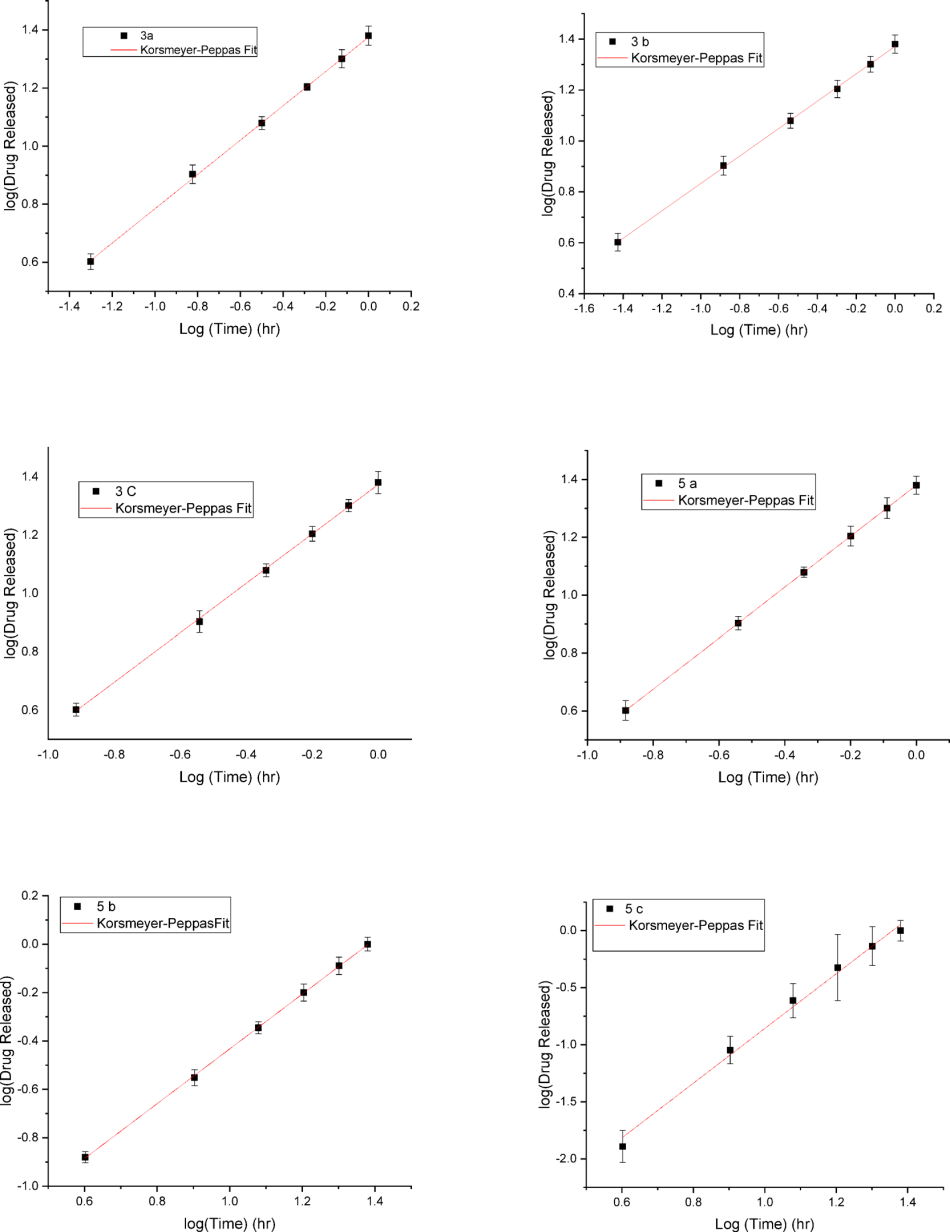
Korsmeyer–Peppas drug release model for samples **3a–c**, **5a–c**: excellent fit with R^2^ values ranging from 0.99 to 1, indicating high correlation between experimental and theoretical data.

Figure S16A, B shows that for the First and Zero Order Fits, the R^2^ values for these fits are generally lower than those for Korsmeyer–Peppas and Higuchi, indicating that these models may not describe the release kinetics as accurately as the others. These findings suggest that the Korsmeyer–Peppas Fig. 3 and Higuchi Fig. S16C models are most suitable for describing the drug release profiles of these compounds, with Korsmeyer-Peppas providing the best fit overall. The Korsmeyer–Peppas model Fig. 3 appears to provide the best fit overall, suggesting that it captures the complexity of the drug release mechanisms more effectively than the other models. The zero-order and Higuchi models are also useful but may not fully describe the complex release behaviors seen in these compounds.

The Korsmeyer–Peppas data reveals distinct patterns in the release kinetics of the compounds, with variations in the release exponent n, intercept, and release rate constant Kn across the different structures. For compounds with n values between 0.54 and 0.88, such as 3a, 3b, and 3c, the release mechanism is governed by anomalous transport, where the release is a combination of diffusion and matrix effects. These compounds exhibit relatively high Kn around 23.99 and 23.44, suggesting faster release rates, which is typical for hydrophilic compounds that dissolve more readily in PBS. In contrast, for compounds **5b**, and **5c**, n values greater than 1 indicate a shift towards super case II transport. The lower Kn values for these compounds suggest slower, more controlled release profiles, which can be attributed to the presence of hydrophobic groups. These groups reduce the solubility of the compounds in PBS, resulting in a sustained release rather than rapid dissolution.

The surface morphology of thin films plays a pivotal role in dictating drug release kinetics. Enhanced surface roughness increases the surface area, facilitating greater interaction with the release medium and potentially accelerating drug diffusion. Studies have demonstrated that rougher surfaces can improve drug release rates due to this increased interaction^84^. Additionally, the porosity of a film influences its drug release behavior; higher porosity provides more pathways for drug diffusion, leading to faster release profiles^85^. Furthermore, the chemical composition and proportion of polymers used in film formation affect surface properties and dissolution rates. Variations in composition can alter surface energy and hydrophilicity, impacting drug release mechanisms.

#### Setting up cell cultures on experimental films

**Fig. 4:**
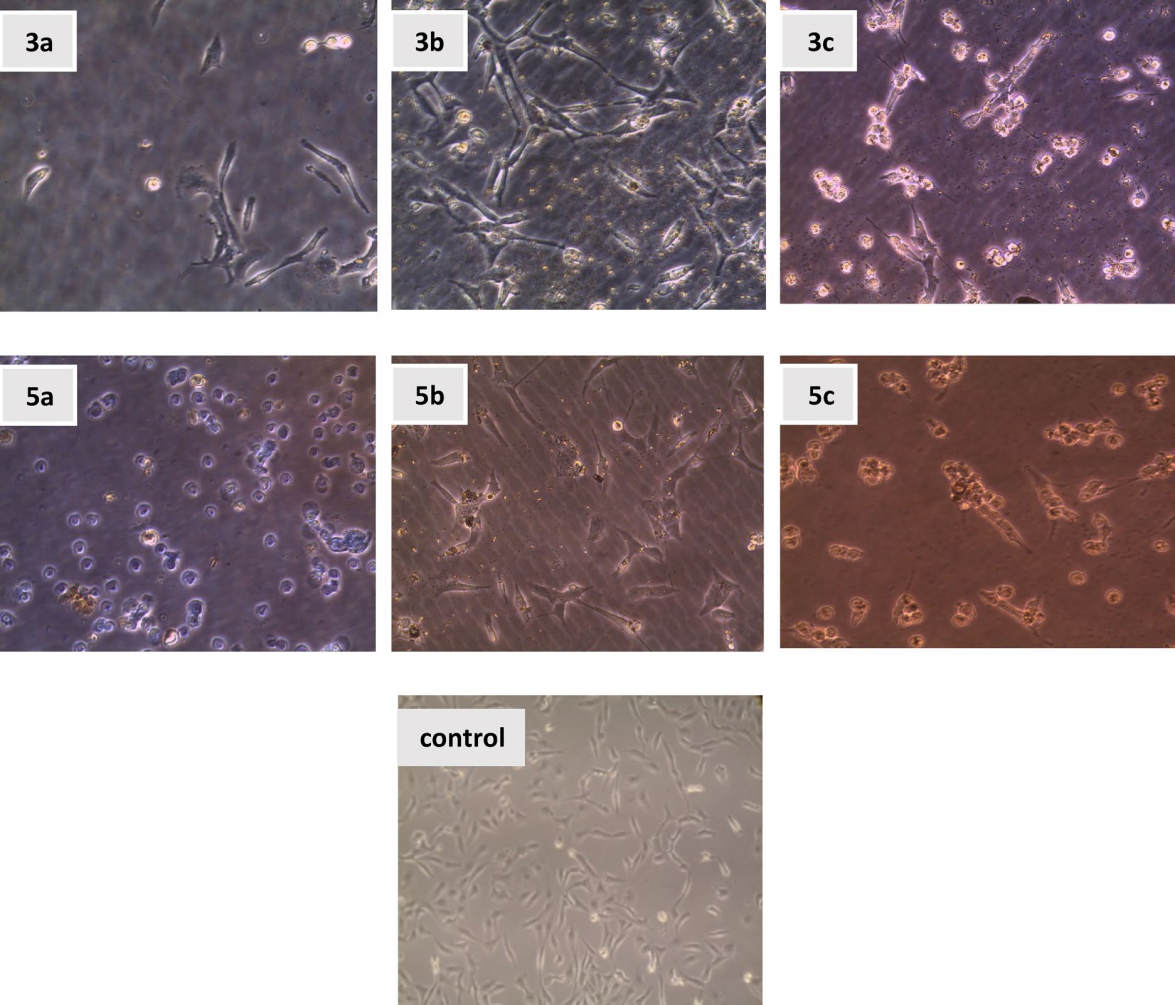
Displays optical micrographs captured using an inverted microscope, showing HepG-2 cancer cells on various substrates: (**a**) **3a–c** and **5a–c**, rounded, detached cells are often indicative of dying cells, while those maintaining the typical elongated shape are likely still alive, control cells: (**b**) morphology and distribution of HepG-2 cells without substrate intervention.

Figure 4 represents typical optical micrographs of living HepG2 cancer cells obtained using an inverted microscope. The cell lines displayed an epithelial morphology and adherent properties in cell culture on bare glass. As shown in Fig. 4, our results revealed a reduction in the number of cancer cells on the THIO-coated surfaces. The number of cells on the THIO-coated surfaces (**3a–c**) is even lower than on the bare glass surfaces. The cells on THIO-coated surfaces (**5a–c**) exhibited apoptotic and necrotic behavior compared with the control and THIO-coated surfaces (**3a–c**).

In control cells, the cells appear well-distributed with typical morphology seen in healthy HepG-2 cells. They are elongated and spindle-shaped, indicating that they are alive and attached to the substrate. The even distribution and healthy appearance suggest that the control cells are thriving under standard culture conditions. In **3a**, the cells in this image appear more irregular in shape compared to the control. Some cells seem rounded and detached from the surface, which is often a sign of cell death or stress. In **3b**, there is a mix of cell morphologies. Some cells retain a more elongated form, while others appear more rounded and darker, which may indicate that some cells are alive but stressed, while others are undergoing cell death. In **3c**, the HepG2 cells cultured on this substrate exhibit typical epithelial morphology but may show slightly less attachment and proliferation compared to the control cells. In **5a** The HepG2 cells on this substrate could display increased apoptosis or reduced proliferation. In **5b**, HepG2 cells on this surface might exhibit pronounced apoptotic and necrotic. In **5c**, HepG2 are likely more detached or dead, displaying features of both apoptosis and necrosis.

There is moderate impact on cell adhesion and proliferation due to the hydrophobic nature of the methyl group in methyl derivatives **3a**,** 5a**, cells show apoptotic behavior. Cells may be rounded and exhibit early signs of apoptosis. While, methoxy derivatives **3b**,** 5b**, show typical epithelial morphology, but potentially fewer in number compared to the control. Chloride Derivatives **3c**,** 5c**, show the strongest impact on cell viability^86^. The chemical groups (methyl, methoxy, and chloride) modulate the surface’s polarity and hydrophobicity, which in turn influences how HepG2 cells adhere, proliferate, and survive. The films significantly reduced HepG2 liver cancer cell adhesion (~ 78% decrease vs. control).

**Fig. 5:**
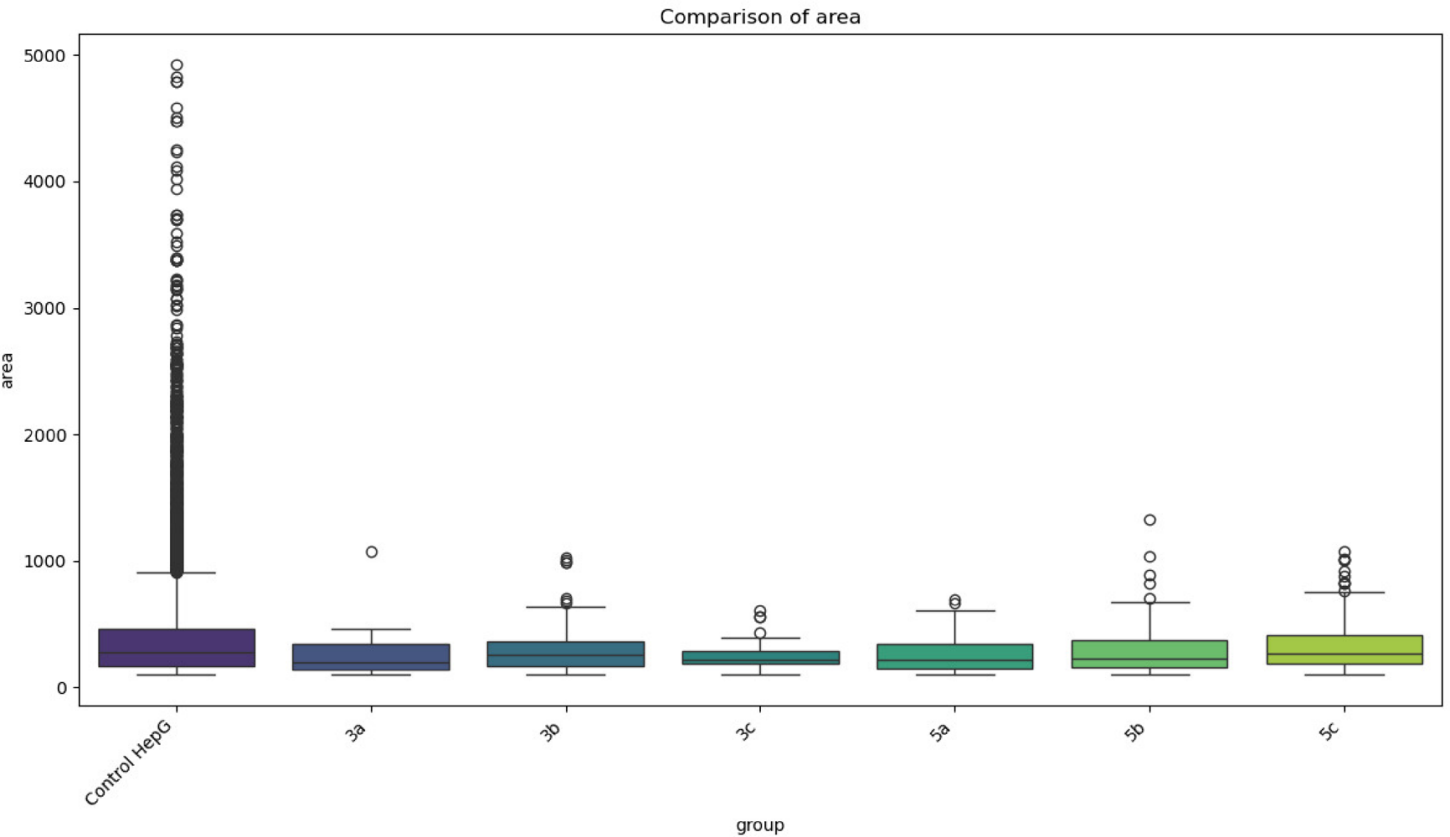
Boxplot comparison of cell area across the control and treated groups. Cell area (µm^2^), vs. experimental groups. The control group shows the highest variability and extreme outliers, while all treated groups exhibit reduced spread and fewer outliers, indicating more uniform cell sizes post-treatment.

The boxplot Fig. 5, illustrates a comparison of cell area between the control group HepG2 cell and THIO-coated surfaces (**3a**,** 3b**,** 3c**,** 5a**,** 5b**,** 5c**). The control group displays a significantly wider range of area values, with numerous high outliers extending up to approximately 4900 μm^2^, indicating substantial variability and the presence of enlarged cells under normal conditions. The median area in the control group is around 350 μm^2^, but the large spread and high number of outliers reflect natural heterogeneity. In contrast, all THIO-coated surfaces exhibit a notably narrower distribution of cell area, with median values clustered between approximately 250 and 350 μm^2^. These THIO-coated surfaces also show fewer and smaller outliers, suggesting a more consistent cell size following treatment. **3b** shows a slightly wider spread than others, with some outliers reaching around 1000 μm^2^, whereas THIO-coated surface 3a presents a tighter distribution with a lower median. **5a**, **5b**, and **5c** show relatively similar patterns, but 5c appears to have a slightly higher median and more upper-range outliers than **5a** or **5b**.

The potential application of THIO thin films in implantable medical devices lies in their ability to reduce cancer cell adhesion and enhance controlled drug release. To adapt these films for biomedical use, it is critical to optimize their biocompatibility, mechanical stability, and surface modification capabilities. By leveraging these characteristics, THIO thin films could be engineered to minimize biofouling, correct tissue compatibility, and increase the functional lifespan of implantable devices. Such innovations would contribute to the development of next-generation biomedical implants that maintain performance and biostability in physiological environments.

### Theoretical calculations

#### In silico-target prediction

**Fig. 6:**
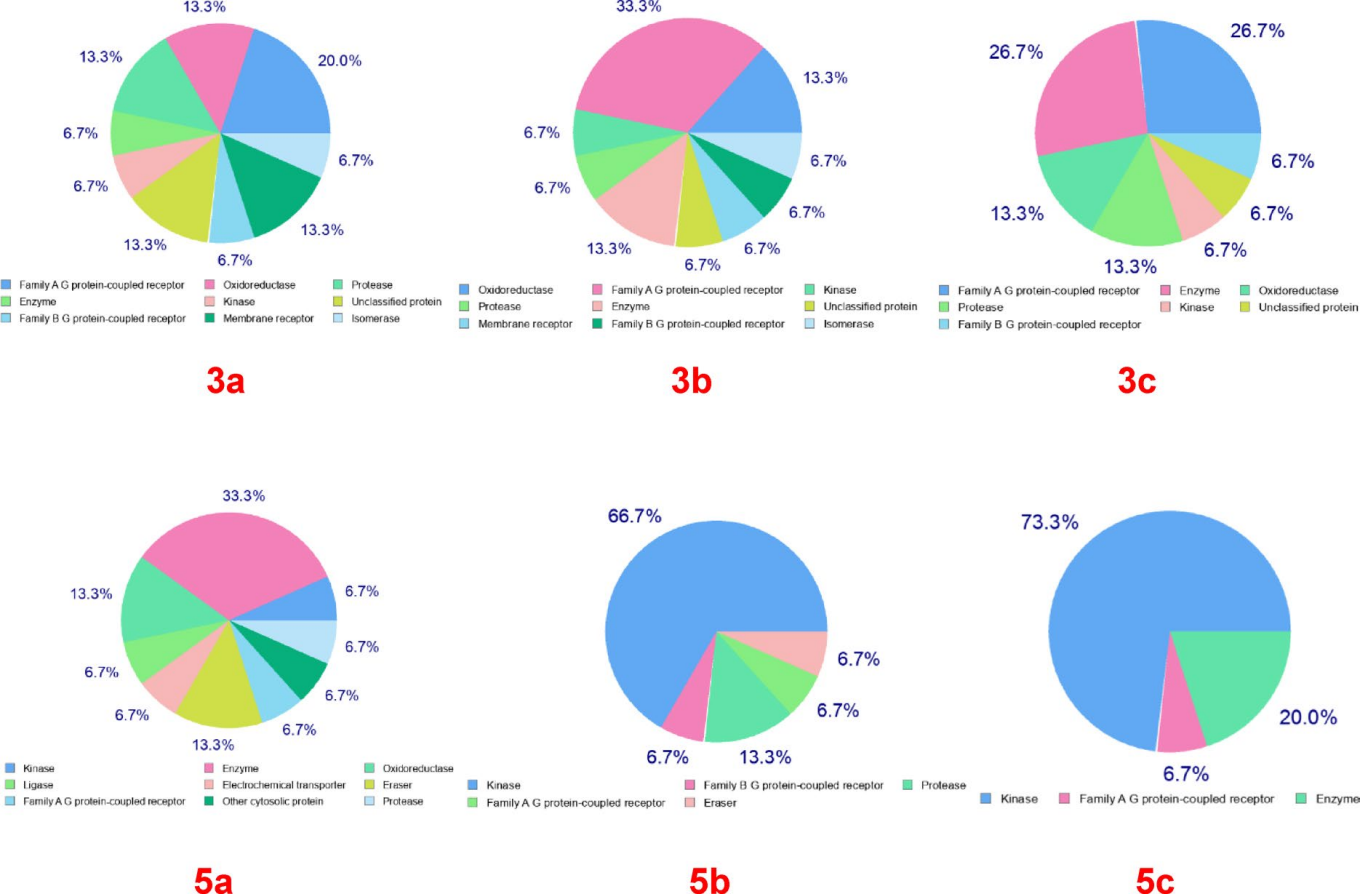
Swiss target prediction scanning for compounds **3a–c**, **5a–c**.

The Swiss Target Prediction software was used to scan the novel compounds **3a–c** and **5a–c**, revealing their notable potential to bind with kinases, especially **protein tyrosine kinase**, as depicted in (Fig. 6). Compounds **5b** and **5c** exhibited the highest kinase affinities of 66.7% and 73.3%, respectively, whereas compound **3c** showed a kinase affinity of 26.7%. Additionally, the kinase affinities for compounds **3a**, **3b**, and **5a** were recorded at 20%, 13.3%, and 6.7%, respectively. PPI Network Analysis and Functional Enrichment was used to express the functional categories of significantly altered proteins in HEpg2 cells (Fig. S17). Janus kinase 1 (JAK1) is a critical component of the JAK-STAT signaling pathway, playing a key role in mediating the effects of various cytokines and growth factors. In the interleukin-2 (IL-2) pathway, JAK1, along with JAK3, is activated upon IL-2 binding to its receptor, leading to T cell proliferation and differentiation. Similarly, in the interferon-gamma (IFN-γ) pathway, JAK1 partners with JAK2 to phosphorylate STAT1, driving immune responses. JAK1 also collaborates with JAK2 in the interleukin-6 (IL-6) pathway, where it activates STAT3 to mediate inflammation and immune responses. Furthermore, JAK1 is essential in the type I interferon (IFN) signaling pathway, working with TYK2 to phosphorylate STAT1 and STAT2, forming the ISGF3 complex that induces antiviral gene expression. In the growth hormone receptor (GHR) pathway, JAK1 assists JAK2 in phosphorylating STAT proteins to regulate growth-related genes^87^.

### Molecular docking

The interesting target-affinity prediction of our compounds to protein tyrosine kinase enzyme was further investigated using molecular docking studies. Table 3, shows docking score, root mean square deviation RMSD and the ligand interactions with PDB 4E4L. The amino acids involved in the interaction were Arg 877, Arg1007, Glu 1033, Glu 883, Lys 876, Ile 878, Tyr 1059, Ser 1137, Asp 1021.

**Fig. 7:**
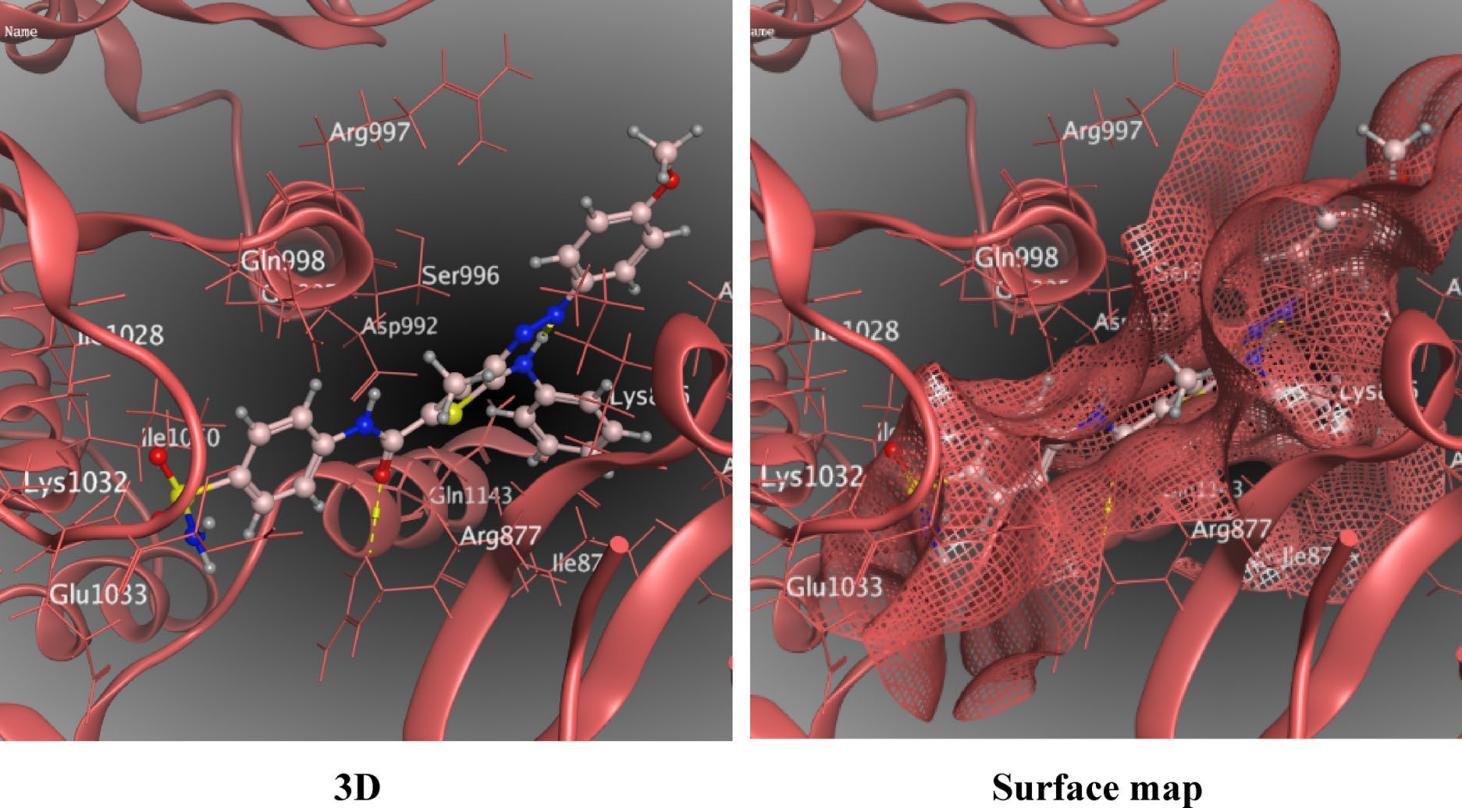
The interaction between 3-methylthiophene **5b** and (PDB ID: 4e4l).

These interactions were either hydrogen bonding (donor or acceptor) or hydrophobic interactions (π –H interaction). The interaction through the amino acid Arg 877 was a common feature between the synthesized compounds and the active ligand site, as primarily depicted.in Table 3. Sorafenib as standard drug was sharing the same amino acid interaction. Compound **5b** as 3-methyl thiophene showed the best binding energy to the pocket (7.59 kcal/mol) and it also showed a remarkable apoptosis behavior against HepG2 cells, Fig. 7, S18–S23. Also compounds **5a** and **5c** having the docking scores (6.48 and 6.59 kcal/mol) shares the uniqe apoptosis performance against HepG2 cells. Compound **5c** expressed the lowest RMSD value, with 0.73 A. 3-hydroxy thioehene derivatives **3a–c** exhibited docking scores 6.58, 6.55, 6.44 kcal/mol, respectively. They also share similar behaviour on HepG2 cells.

**Table 3:**
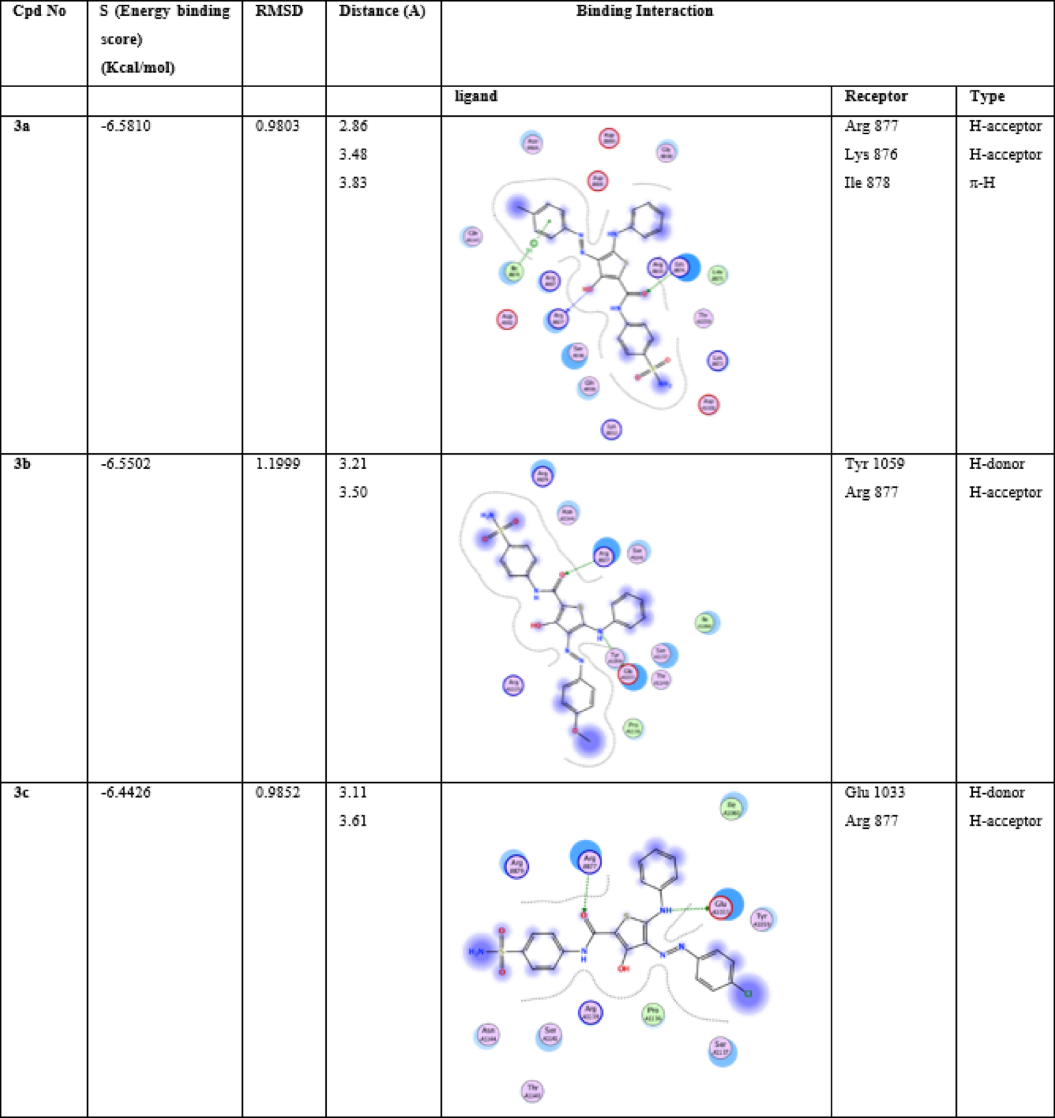

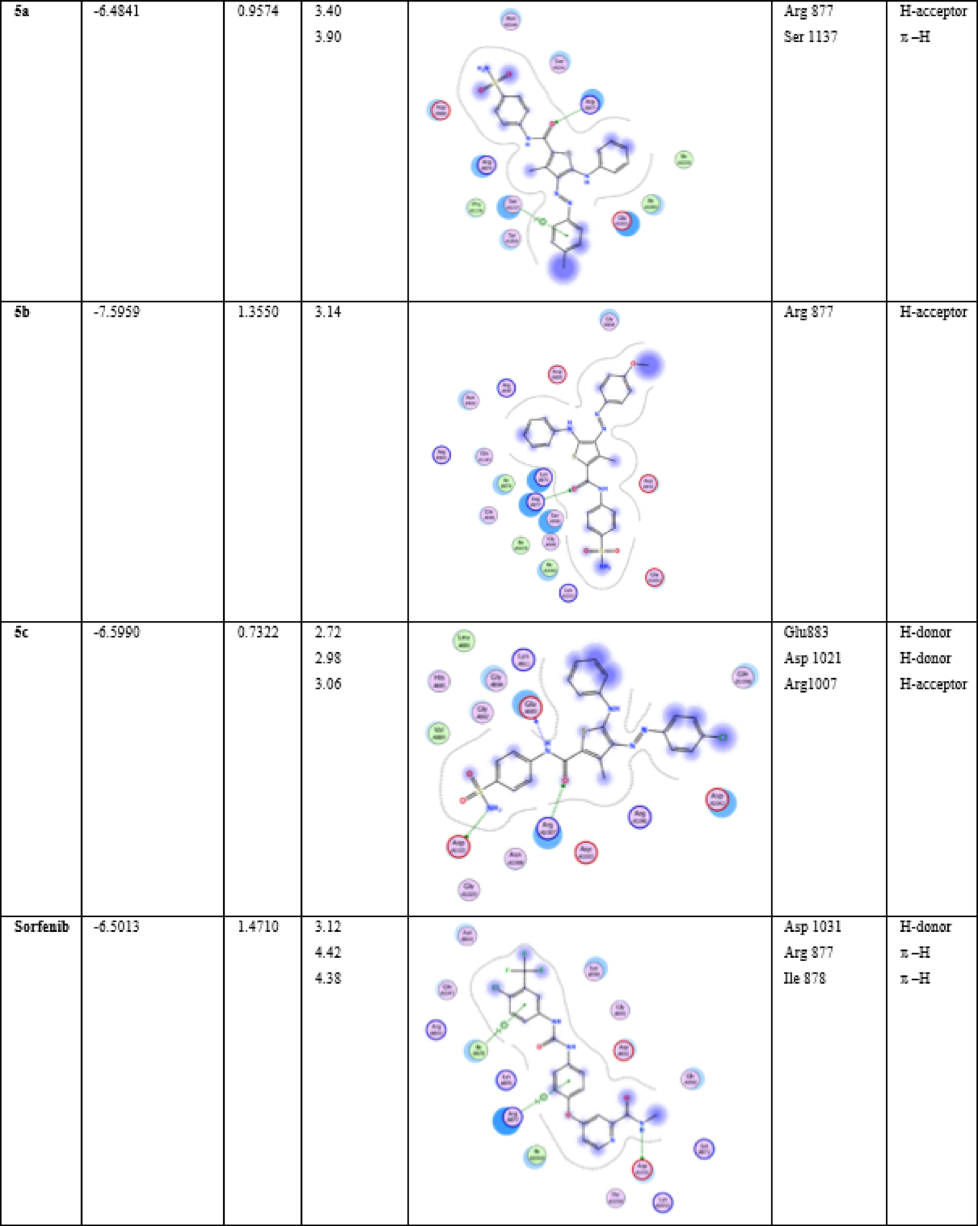
Interaction between ligands and protein **4E4L**, showing energy of binding, RMSD, 2D structure, interaction type and distance with specific receptor.

#### DFT-calculation

**Fig. 8:**
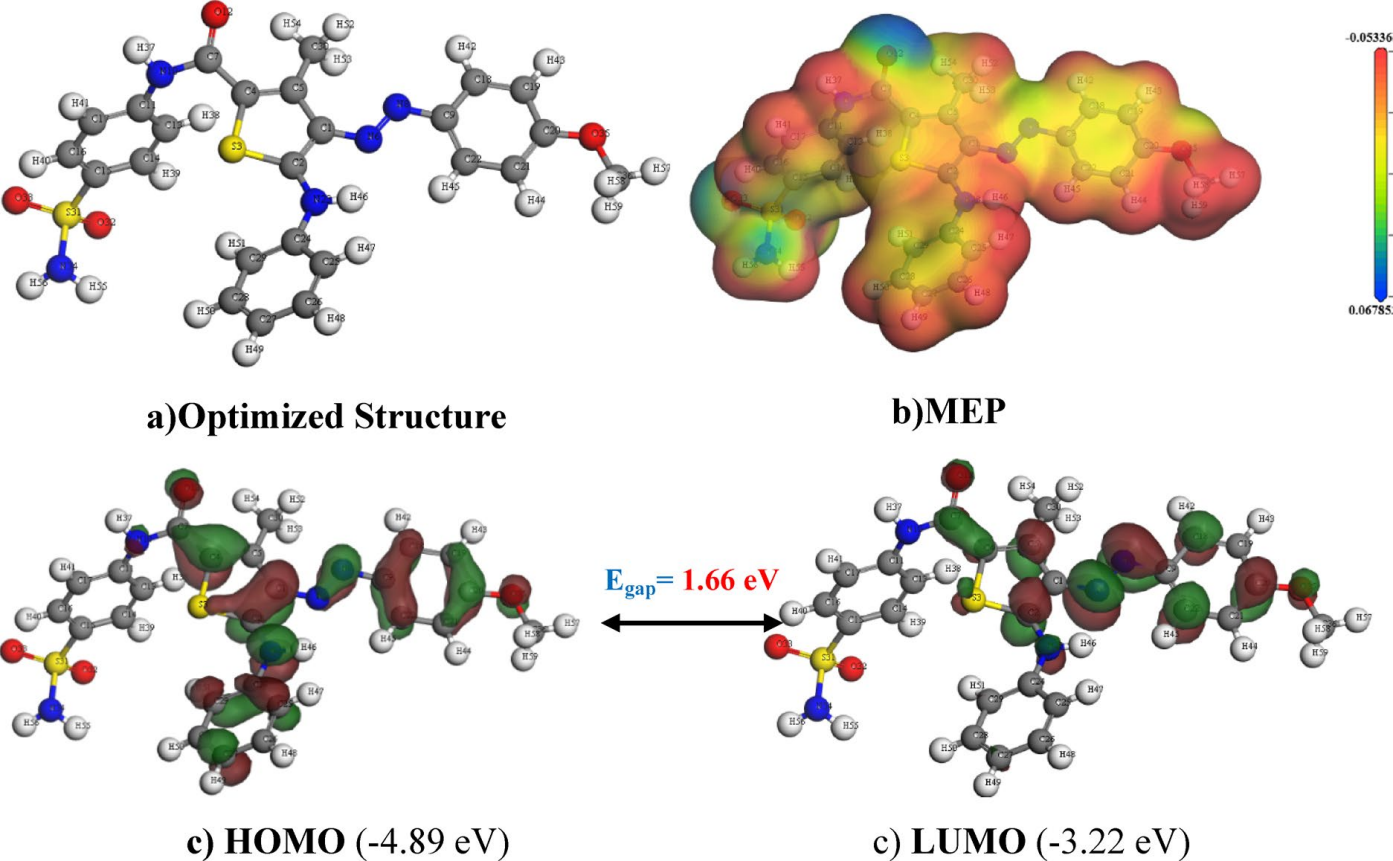
(**a**) Optimized structures, (**b**) electron density, (**c**) HOMO and LUMO for compound **5b**.

To gain insight into electronic properties and chemical reactivity of investigated compounds, computational method is utilized to analyze frontier molecular orbitals (FMOs) and molecular electrostatic potential (MEP). The optimized structures and the numbering of atoms of **3a–c** and **5a–c** are shown in Fig. 8, S24–S28. Where calculated isodensities of LUMO (lowest unoccupied molecular orbital) and HOMO (highest occupied molecular orbital) of the investigated molecules are presented in Table 4. These parameters were indicative of their electron accepting and donating nature, respectively. The HOMO-LUMO energy gap, ΔE_H−L_ = E_H_–E_L_, and MEP plots illustrates the molecule’s chemical stability, charge transfer and reactive sites^88,89^. Molecule bioactivity claims a decrease of HOMO-LUMO energy gap validating more ease charge transfer interactions within the molecule^90^. Also easier HOMO-LUMO electron transfer by absorbing light energy of a specific wavelength^91^ and recent studies offer valuable comparative frameworks^92,93^. The energy levels of HOMO and LUMO and ΔE_H−L_ was theoretically estimated in Table 4.

The HOMO plots of **3a**, **5a** consists of the non-bonding lone pair of heteroatoms π-orbitals of *N*-C-O carboxamide group, *N*-phenyl moiety except two carbons, thiophene carbons except C-OH or C-Me, azophenyl moiety except two carbons and 4-methyl substituent. The HOMO plots of **3b**, **5b** consists of the non-bonding lone pair of heteroatoms π-orbitals *N*-C-O carboxamide group, *N*-phenyl moiety except two carbons, thiophene carbons except C-OH or C-Me, azophenyl moiety except 4-methyl substituent. The HOMO plots of **3c**,** 5c** consists of the non-bonding lone pair of heteroatoms π-orbitals *N*-C-O carboxamide group, *N*-phenyl moiety except two carbons, thiophene carbons except C-OH, azophenyl moiety except two carbons. While, the LUMO plots of **3a-c** consists of the non-bonding lone pairs of *N-*aniline, π-orbitals of thiophene carbons except C-carboxamide group, azophenyl moiety except two carbons and (4-methyl substituent or Cl gp). The LUMO plots of **5a-c** consists of the non-bonding lone pairs of *N-*aniline, π-orbitals of thiophene ring except C-Azo, C-O carboxamide group, azophenyl moiety except two carbons and (4-methyl substituent or Cl gp).

The observed interactions were reflected on the E_H_ and E_L_ values as the E_H_ of all compounds, ranging closely between − 4.89 and − 5.24 eV, while E_L_ values between − 3.22 and − 3.46 eV. Among these, the methyl derivative **5b** exhibited the lowest E_H_ value compared to its counterparts, suggesting a ranking of the hydroxy and methyl derivatives from highest to lowest as **b** > **a** > **c** based on their E_H_ and E_L_ values. Furthermore, the HOMO-LUMO energy gap data, ΔE_H−L_, ranging from 1.66 to 1.78 eV, indicated that the hydroxy derivative **3c** had the highest gap value among both the hydroxy and methyl groups, **3a–c** and **5a–c** respectively, with the hydroxyl derivatives generally exhibiting higher values than the corresponding methyl derivatives. The values of ΔE_H−L_, for the hydroxy and methyl derivatives ordered **b** > **a** > **c**. Finally, the hydroxy derivatives, **3c**, showed higher E_HOMO_ and ΔE_H−_L while lower E_LUMO_ than other derivatives, while methyl derivative **5b**, showed the lowest E_HOMO_, E_LUMO_ and ΔE_H−L_, which is clearly has shown curiously as apoptotic behavior against HepG2 cells. Recent studies^93–95^highlighted the role ΔE_H−L_ influencing chemical reactivity and docking behavior, which aligns with our findings for compound **5b**. The MEP surface map Fig. 8b, provides a comprehensive visualization of the compound **5b** electrostatic potential as follow; the red region (e.g., near N and O) indicate electron-rich regions, conductive for electrophilic interactions. While blue region indicate electron-poor sites, which are suitable for nucleophilic reactions or hydrogen bonding.

**Table 4:** The HOMO energy (E_HOMO_), LUMO energy (E_LUMO_), HOMO–LUMO energy gap (ΔE_H−L_) in eV.

Compound	HOMO eV	LUMO eV	ΔE_H−L_
**3a**	− 5.09	− 3.32	1.77
**3b**	− 4.99	− 3.22	1.77
**3c**	− 5.24	− 3.46	1.78
**5a**	− 4.97	− 3.30	1.68
**5b**	− 4.89	− 3.22	1.66
**5c**	− 5.15	− 3.45	1.69

## Conclusion

This study successfully modifies glass substrate surfaces using six novel thiophene-based (THIO) coatings to minimize cancer cell adhesion and enable controlled drug release. The results highlight the potential of THIO coatings in improving the biocompatibility and functionality of medical implants by reducing biofouling and lowering post-surgical risks. Cytotoxicity analysis demonstrated that different THIO derivatives exhibit distinct biological effects: methyl derivatives moderately affect HepG2 cell adhesion and proliferation, methoxy derivatives significantly induce apoptosis due to their electron-donating properties and hydrophilicity, while chloride derivatives exert the strongest cytotoxic effects by disrupting cellular ionic balance and triggering apoptosis and necrosis.

The ability of THIO coatings to regulate drug release and selectively impact cancer cell viability suggests their suitability for targeted biomedical applications, particularly in oncology. Further investigations using transmission electron microscopy (TEM) could provide deeper insights into how THIOs interact with various cancer cell types, aiding in the development of customized thiophene-based therapies. Expanding research to bladder cancer and renal cell carcinoma could help assess the broader effectiveness of these coatings, especially in relation to preventing tumor cell adhesion in biopsy and surgical procedures.

To translate THIO coatings into clinical applications, several critical aspects must be addressed, including biocompatibility, durability, functional performance, large-scale production, and regulatory approval. Before THIO-based coatings can be used in clinical applications, several critical challenges must be addressed. These include ensuring their biocompatibility, long-term stability, and effectiveness in vivo, which would require extensive preclinical testing. Regulatory approval processes, such as those from the FDA or EMA, would need to be navigated, requiring rigorous clinical trials to demonstrate safety and efficacy. Additionally, challenges related to manufacturing scalability, reproducibility, and cost-effectiveness must be addressed to ensure widespread commercialization. Collaborations with industry partners will be essential to overcome these obstacles and bring THIO coatings to market for real-world medical use. Beyond liver cancer applications, the research assesses the potential of THIO coatings in biomedical fields, particularly in implantable medical devices and surgical instruments, by analyzing their cytotoxicity, biocompatibility, and stability. Future investigations will explore their effectiveness in preventing tumor seeding during biopsies and their role in treating bladder cancer and renal cell carcinoma, expanding their applicability in diverse oncological environments. Future research should focus on conducting further in vitro and in vivo studies to validate the selective targeting of cancer cells and explore the long-term safety and efficacy of the approach. Additionally, clinical trials will be essential to determine its potential for translating into a viable treatment option for cancer patients.

## Electronic supplementary material

Below is the link to the electronic supplementary material.

Supplementary Material 1

## Data Availability

The datasets used and/or analyzed during the current study available from the corresponding author on reasonable request.
