# Supplementary material for: Tuning surface properties of thiophene-based thin films on glass substrates for cancer cell adhesion, drug release control, and computational analysis
Source: Sci Rep. 2025 Jun 20;15:20170. doi: 10.1038/s41598-025-05691-w (PMC12181427; doi:10.1038/s41598-025-05691-w)
Supplement: Supplementary file 1 — Supplementary Material 1 [file 41598_2025_5691_MOESM1_ESM.docx]

**Supporting Information**

**Tuning Surface Properties of Thiophene-Based Thin Films on Glass Substrates for Cancer Cell Adhesion, Drug Release Control, and Computational Analysis**

Heba M. Metwally^a^*, Omar M. El-Banna^a^, Ehab Abdel-Latif^a^, Raghda Abo Gabal^c*^

***^a^*** *Department of Chemistry, Faculty of Science, Mansoura University, 35516 Mansoura, Egypt*

***^b^*** *Center of excellence for Genome and Cancer Research, Urology and Nephrology Center, Mansoura University, El Dakhlia, Egypt.*

**List of figures**

[**Figure S1 ^1^H NMR spectrum of compound 3a 3**](#_Toc181474378)

[**Figure S2 ^13^C NMR spectrum of compound 3a 4**](#_Toc181474379)

[**Figure S3 IR spectrum of compound 3a 5**](#_Toc181474380)

[**Figure S4 ^1^H NMR spectrum of compound 3b 6**](#_Toc181474381)

[**Figure S5 IR spectrum of compound 3b 7**](#_Toc181474382)

[**Figure S6 ^1^H NMR spectrum of compound 3c 8**](#_Toc181474383)

[**Figure S7 IR spectrum of compound 3c 9**](#_Toc181474384)

[**Figure S8 ^1^H NMR spectrum of compound 5a 10**](#_Toc181474385)

[**Figure S9 ^13^C NMR spectrum of compound 5a 11**](#_Toc181474386)

[**Figure S10 IR spectrum of compound 5a 12**](#_Toc181474387)

[**Figure S11 ^1^H NMR spectrum of compound 5b 13**](#_Toc181474388)

[**Figure S12 ^13^C NMR spectrum of compound 5b 14**](#_Toc181474389)

[**Figure S13 IR spectrum of compound 5b 15**](#_Toc181474390)

[**Figure S14 ^1^H NMR spectrum of compound 5c 16**](#_Toc181474391)

[**Figure S15 IR spectrum of compound 5c 17**](#_Toc181474392)

[**Figure S16 In vitro release studies (Zero, First, Higuchi Order Fits) 18**](#_Toc181474393)

[**Figure S17 PPI Network Analysis and Functional Enrichment Revealing the Role of Thiophene, PPI network and functional categories of significantly altered proteins in HEpg2 cells 2**](#_Toc181474394)**1**

[**Figure S18 The interaction between 3-hydroxythiophene 3a and (PDB ID: 4e4l) 2**](#_Toc181474395)**2**

[**Figure S19 The interaction between 3-hydroxythiophene 3b and (PDB ID: 4e4l) 2**](#_Toc181474396)**3**

[**Figure S20 The interaction between 3-hydroxythiophene 3c and (PDB ID: 4e4l) 2**](#_Toc181474397)**4**

[**Figure S21 The interaction between 3-methylthiophene 5a and (PDB ID: 4e4l) 2**](#_Toc181474398)**5**

[**Figure S22 The interaction between 3-methylthiophene 5c and (PDB ID: 4e4l)**](#_Toc181474399) **26**

[**Figure S23 The interaction between Sorafenib and (PDB ID: 4e4l)**](#_Toc181474400) **27**

[**Figure S24 Optimized structures, electron density and HOMO & LUMO for compound 3a.**](#_Toc181474401) **28**

[**Figure S25 Optimized structures, electron density and HOMO & LUMO for compound 3b.**](#_Toc181474402) **29**

[**Figure S26 Optimized structures, electron density and HOMO & LUMO for compound 3c. 3**](#_Toc181474403)**9**

[**Figure S27 Optimized structures, electron density and HOMO & LUMO for compound 5a. 3**](#_Toc181474404)**0**

[**Figure S28 Optimized structures, electron density and HOMO & LUMO for compound 5c. 3**](#_Toc181474405)**0**

**Chemistry**

**Experimental general remarks:** Melting points were determined with Gallenkamp melting point apparatus and are uncorrected. The IR was performed on Bruker Invenio D FTIR spectrometer, Germany. ^1^H NMR and ^13^C NMR spectra were recorded DMSO-*d*_6_ as a solvent using JEOL’s spectrometer at 500 MHz using tetramethylsilane (TMS) as internal standard. Chemical shifts are expressed in δ, ppm. ^1^H NMR data are reported in order: multiplicity (br, broad; s, singlet; d, doublet; t, triplet; dd, doublet of doublet; m, multiplet), approximate coupling constant in Hertz, number of protons and type of protons. The purity of the compounds was checked by ^1^H NMR and thin layer chromatography (TLC) on silica gel plates using a mixture of dichloromethane and methanol or petroleum ether and ethyl acetate as eluent. UV lamp was used as a visualizing agent. Mass analyses and elemental analyses were recorded on Thermo DSQ II spectrometer at Faculty of Science, Alazhar University. The ^13^C NMR spectra of compounds **3b**, **3c**, and **5c** are not recorded due to insufficient solubility in most of NMR solvents.

**Spectral Analysis**

**~~
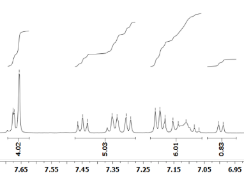

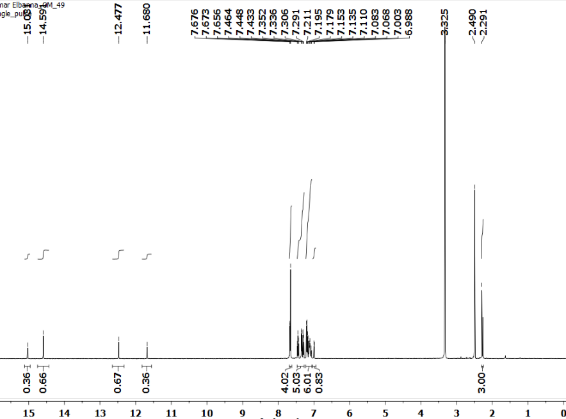
~~**

**Figure S1 ^1^H NMR spectrum of compound 3a**

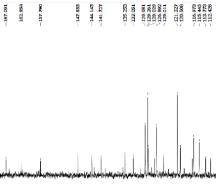

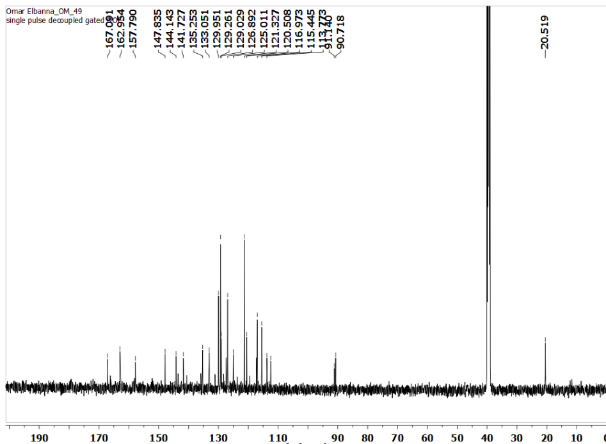


**Figure S2**  **^13^C NMR spectrum of compound 3a**

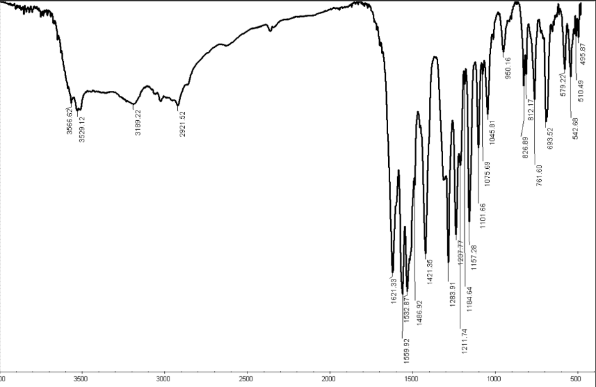


**Figure S3 IR spectrum of compound 3a**

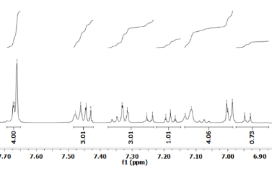

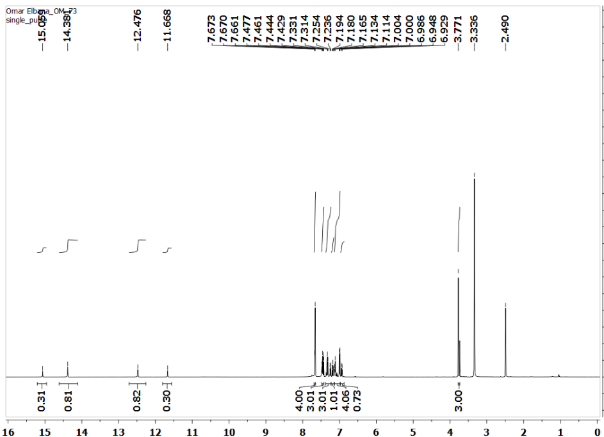


**Figure S4 ^1^H NMR spectrum of compound 3b**

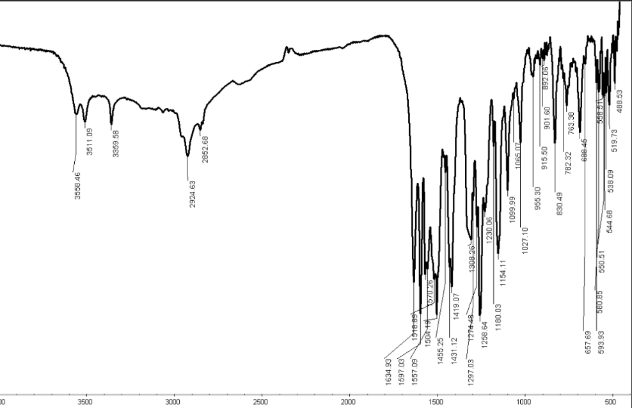


**Figure S5 IR spectrum of compound 3b**

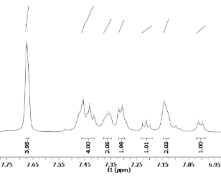
**
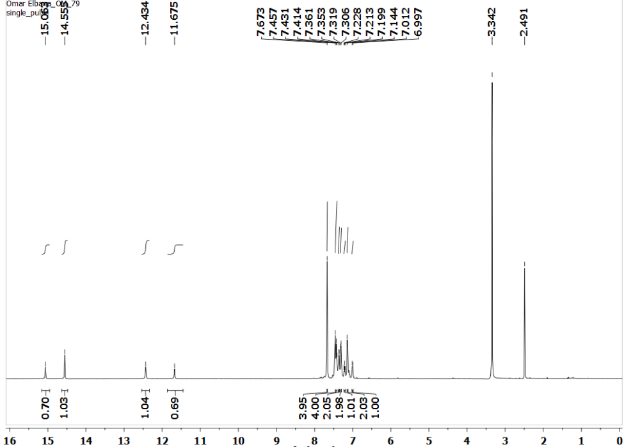
**

**Figure S6 ^1^H NMR spectrum of compound 3c**

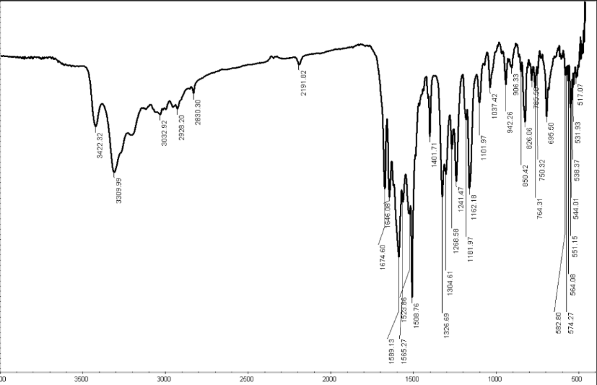


**Figure S7 IR spectrum of compound 3c**

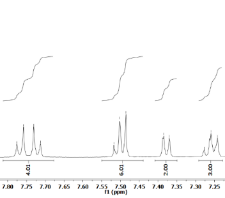

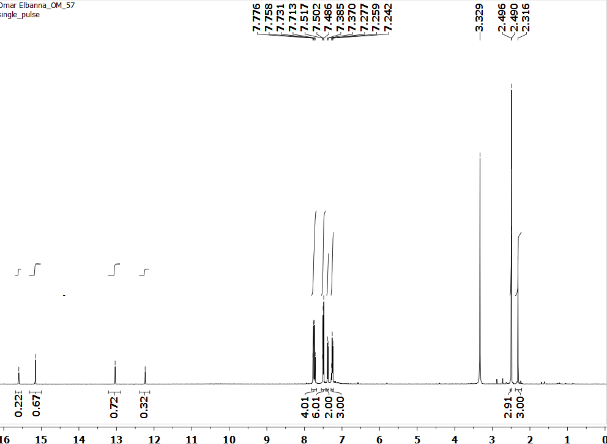


**Figure S8 ^1^H NMR spectrum of compound** **5a**

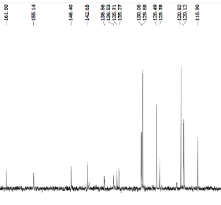

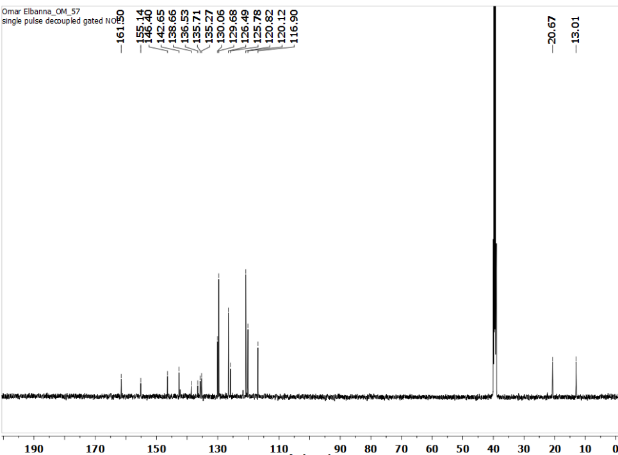


**Figure S9 ^13^C NMR spectrum of compound 5a**

**
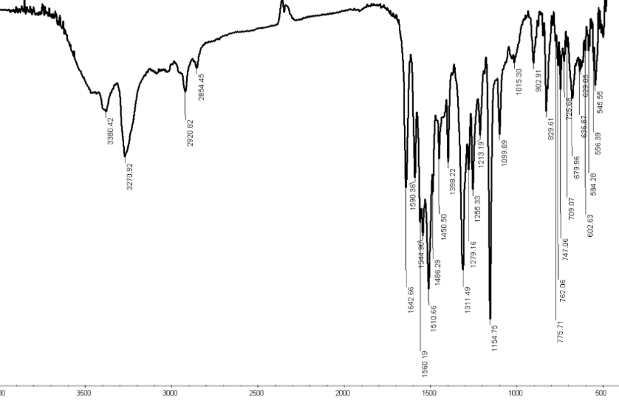
**

**Figure S10 IR spectrum of compound 5a**

**
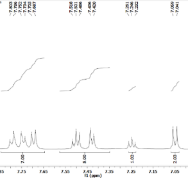

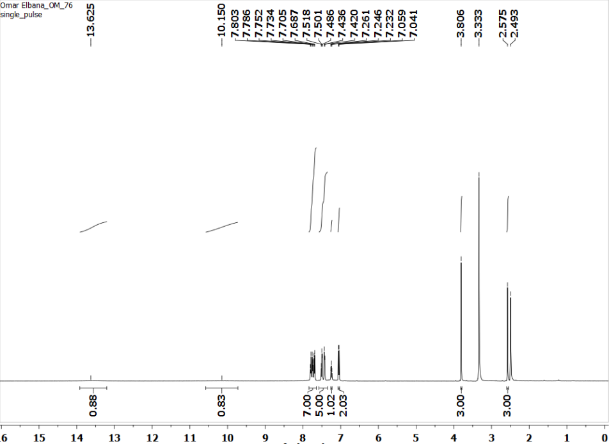
**

**Figure S11 ^1^H NMR spectrum of compound 5b**

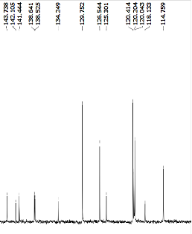

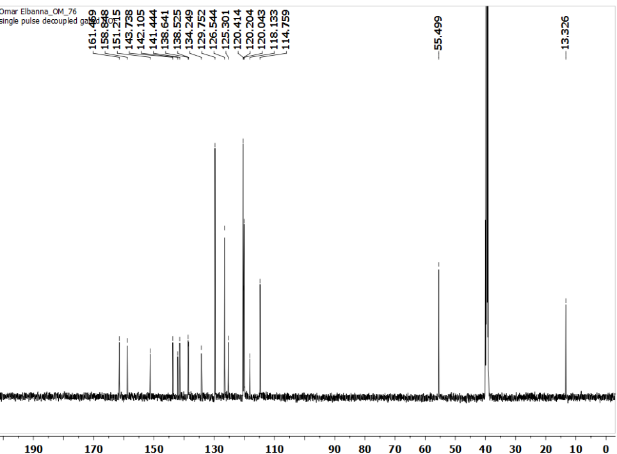


**Figure S12 ^13^C NMR spectrum of compound 5b**

**
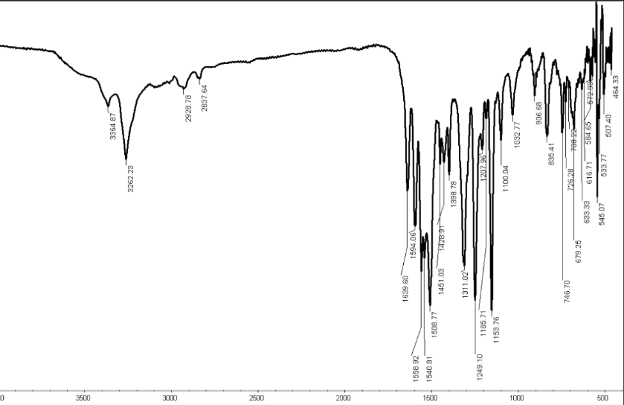
**

**Figure S13 IR spectrum of compound 5b**

**
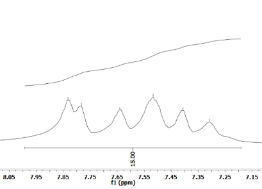

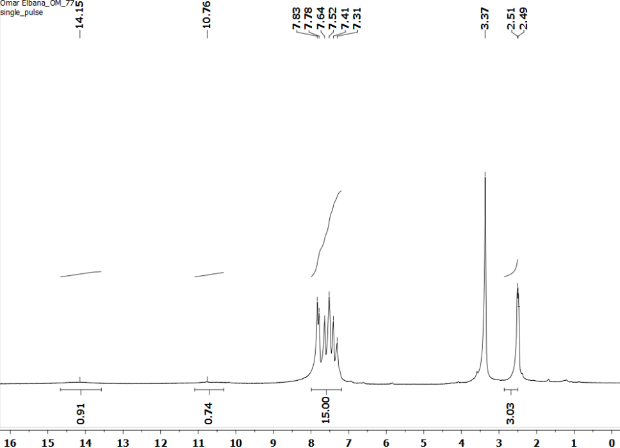
**

**Figure S14 ^1^H NMR spectrum of compound 5c**

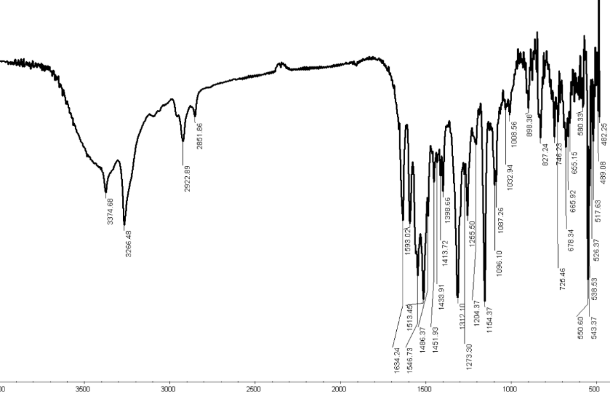


**Figure S15 IR spectrum of compound 5c**

**Figure S16A** Zero-order release kinetics of drug-loaded thin films for compounds **3a, 3b, 3c, 5a**, **5b**, and **5c**. The cumulative amount of drug released (µg) is plotted against time (h), and the linearity of the plots indicates the extent to which each compound follows zero-order release behavior*.*

|  |  |
| --- | --- |
|  |  |
|  |  |
|  |  |
|  |  |

**Figure S16B**First-order release kinetics of drug-loaded thin films for compounds **3a, 3b, 3c, 5a**, **5b**, and **5c**. The logarithm of the remaining drug concentration is plotted against time (h), demonstrating the extent to which the release follows a concentration-dependent (first-order) kinetic model

|  |  |
| --- | --- |
|  |  |
|  |  |
|  |  |
|  |  |

| **Figure.S.16C** Higuchi release kinetics of drug-loaded thin films for compounds **3a, 3b, 3c, 5a**, **5b**, and **5c**. The cumulative amount of drug released (µg) is plotted against the square root of time, indicating diffusion-controlled drug release behavior consistent with the Higuchi model. | |
| --- | --- |
|  |  |
|  |  |
|  |  |
|  |  |
|  |  |
|  | |

**Network analysis of protein interaction data**


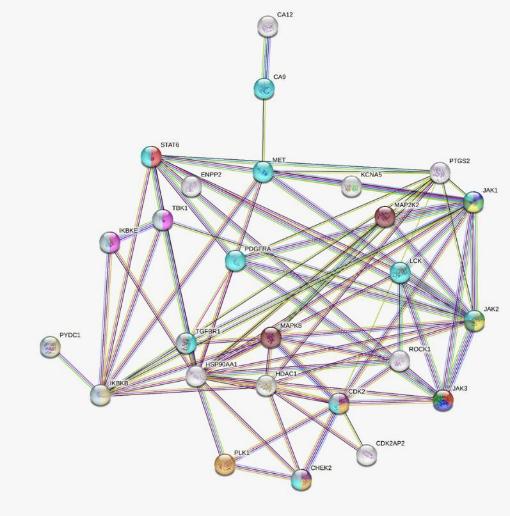


**Figure S17** PPI Network Analysis and Functional Enrichment Revealing the Role of Thiophene, PPI network and functional categories of significantly altered proteins in HEpg2 cells

| 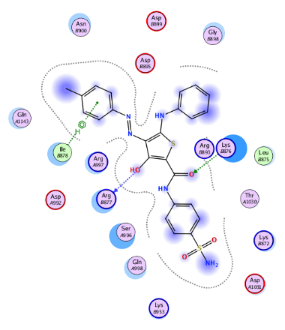 | 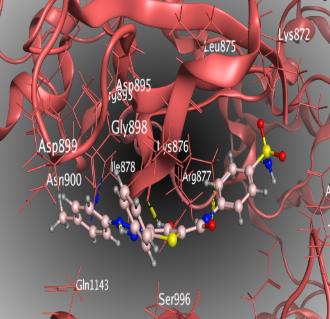 |
| --- | --- |
| **2D** | **3D** |
| **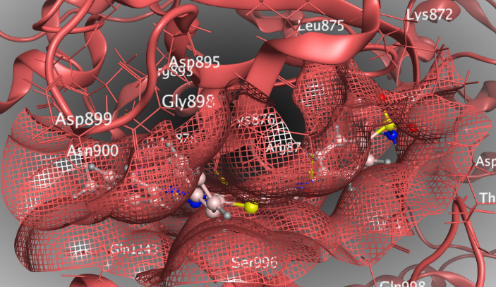** | |
| **Surface map** | |

**Docking (PDB ID: 4e4l)**

**Figure S18** The interaction between 3-hydroxythiophene **3a** and (PDB ID: 4e4l)

| 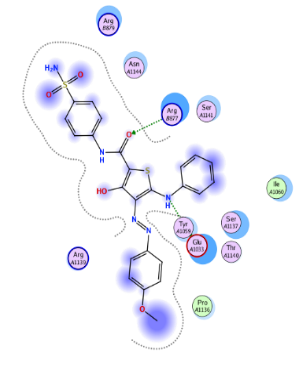 | 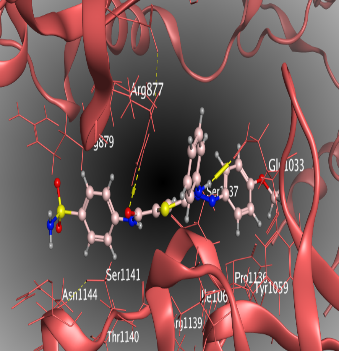 |
| --- | --- |
| **2D** | **3D** |
| **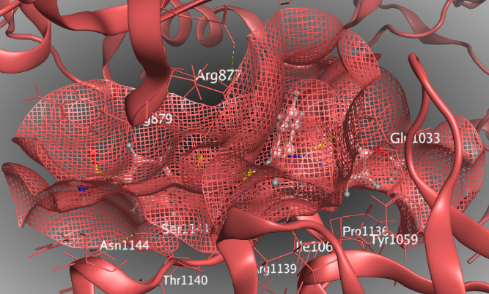** | |
| **Surface map** | |

**Figure S19** The interaction between 3-hydroxythiophene **3b** and (PDB ID: 4e4l)

| 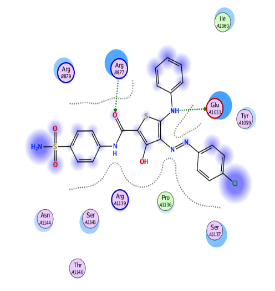 | 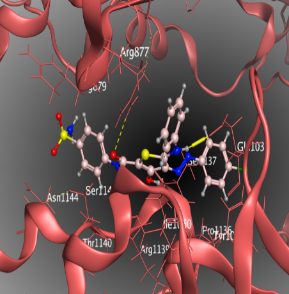 |
| --- | --- |
| **2D** | **3D** |
| **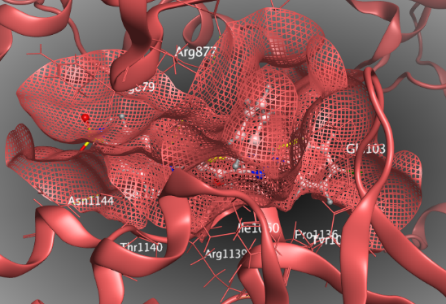** | |
| **Surface map** | |

**Figure S20** The interaction between 3-hydroxythiophene **3c** and (PDB ID: 4e4l)

| 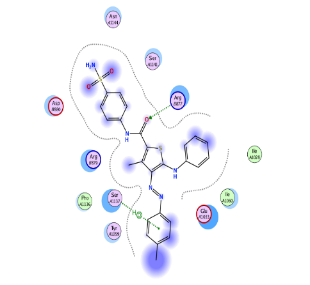 | 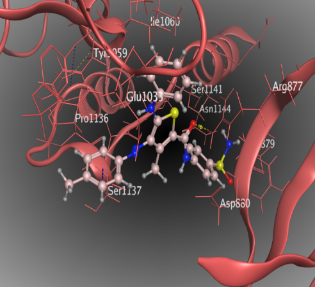 |
| --- | --- |
| **2D** | **3D** |
| **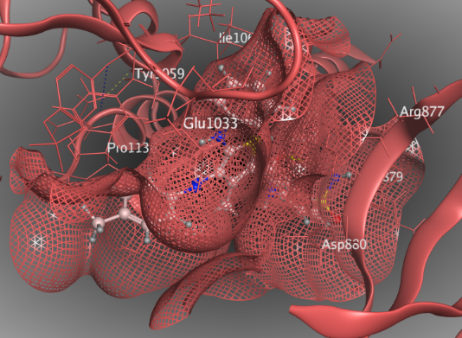** | |
| **Surface map** | |

**Figure S21** The interaction between 3-methylthiophene **5a** and (PDB ID: 4e4l)

| 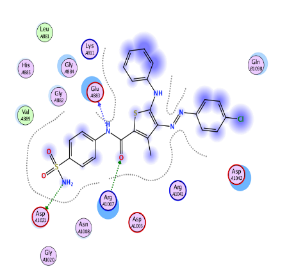 | 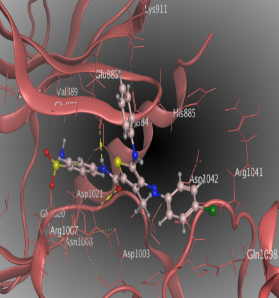 |
| --- | --- |
| **2D** | **3D** |
| **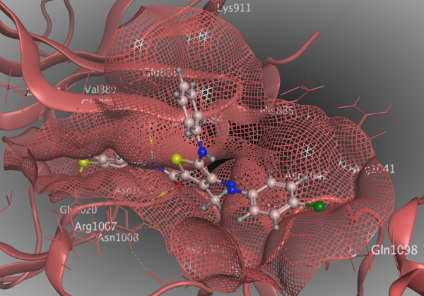** | |
| **Surface map** | |

**Figure S22** The interaction between 3-methylthiophene **5c** and (PDB ID: 4e4l)

| 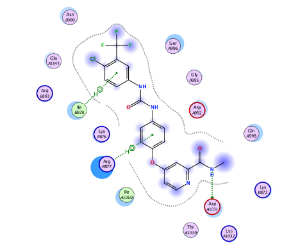 | 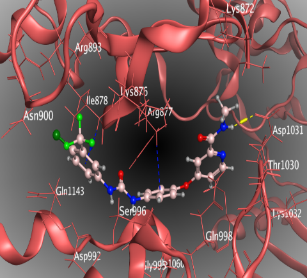 |
| --- | --- |
| **2D** | **3D** |
| **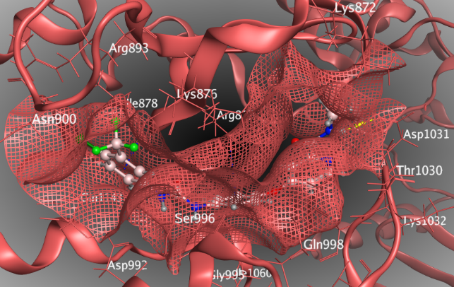** | |
| **Surface map** | |

**Figure S23** The interaction between Sorafenib and (PDB ID: 4e4l)

**DFT-Calculations**

| 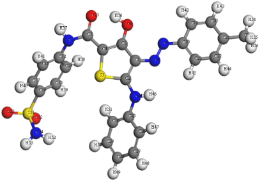  **Optimized Structure** | **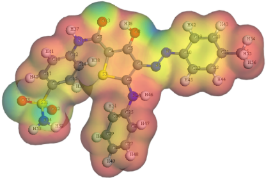MEP** | | |
| --- | --- | --- | --- |
| 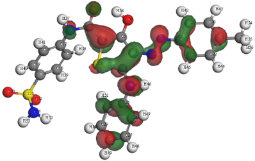 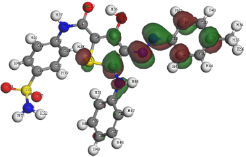  **E_gap_= 1.77 eV**  **HOMO** (-5.09 eV) **LUMO** (-3.32 eV) | | | |
| **Figure S24** Optimized structures, electron density and HOMO & LUMO for compound **3a**. | | | |
| 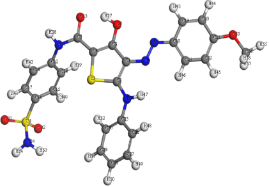  **Optimized Structure** | **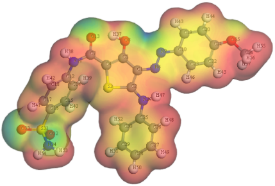**  **MEP** | | |
| **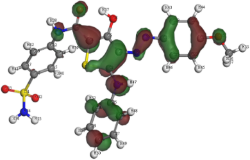** 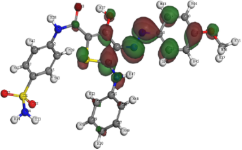  **E_gap_= 1.77eV**  **HOMO** (-4.99 eV) **LUMO** (-3.22 eV) | | | |
| **Figure S25** Optimized structures, electron density and HOMO & LUMO for compound **3b**. | | | |
| 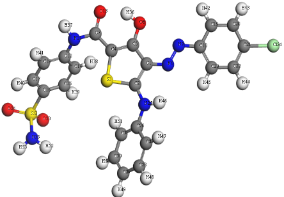  **Optimized Structure** | | | **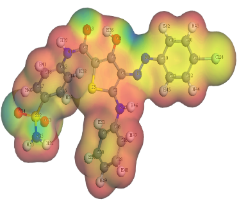**  **MEP** |
| **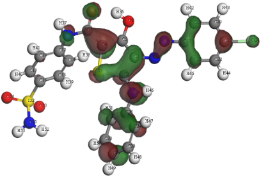** 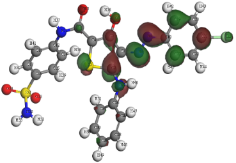  **E_gap_= 1.78 eV**  **HOMO** (-5.24 eV) **LUMO** (-3.46 eV) | | | |
| **Figure S26** Optimized structures, electron density and HOMO & LUMO for compound **3c**. | | | |
| 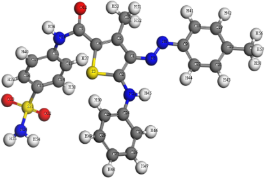  **Optimized Structure** | **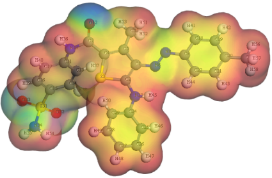MEP** | | |
| 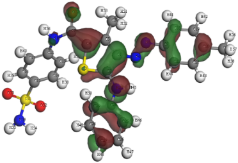 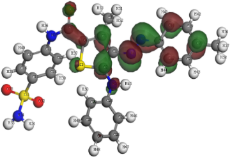  **E_gap_= 1.68 eV**  **HOMO** (-4.97 eV) **LUMO** (-3.30 eV) | | | |
| **Figure S27** Optimized structures, electron density and HOMO & LUMO for compound **5a**. | | | |
| 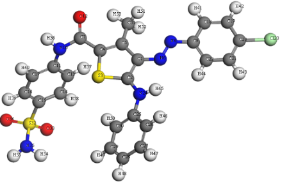  **Optimized Structure** | | **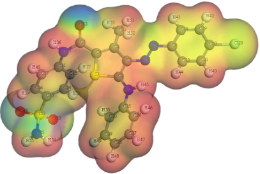**  **MEP** | |
| **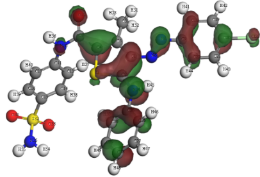** 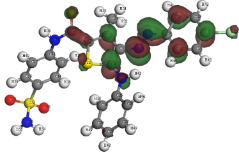  **E_gap_= 1.69 eV**  **HOMO** (-5.15 eV) **LUMO** (-3.45 eV) | | | |
| **Figure S28** Optimized structures, electron density and HOMO & LUMO for compound **5c**. | | | |
